# Genital Herpes Has Played a More Important Role than Any Other Sexually Transmitted Infection in Driving HIV Prevalence in Africa

**DOI:** 10.1371/journal.pone.0002230

**Published:** 2008-05-21

**Authors:** Laith J. Abu-Raddad, Amalia S. Magaret, Connie Celum, Anna Wald, Ira M. Longini, Steven G. Self, Lawrence Corey

**Affiliations:** 1 Statistical Center for HIV/AIDS Research and Prevention, Fred Hutchinson Cancer Research Center, Seattle, Washington, United States of America; 2 Center for Studies in Demography and Ecology, University of Washington, Seattle, Washington, United States of America; 3 Program in Biostatistics, Fred Hutchinson Cancer Research Center, Seattle, Washington, United States of America; 4 Department of Medicine, University of Washington, Seattle, Washington, United States of America; 5 Department of Epidemiology, University of Washington, Seattle, Washington, United States of America; 6 Virology Research Clinic, University of Washington, Seattle, Washington, United States of America; 7 Department of Laboratory Medicine, University of Washington, Seattle, Washington, United States of America; 8 Program in Biostatistics and Biomathematics, Fred Hutchinson Cancer Research Center, Seattle, Washington, United States of America; 9 Department of Biostatistics, University of Washington, Seattle, Washington, United States of America; 10 Clinical Research Division, Fred Hutchinson Cancer Research Center, Seattle, Washington, United States of America; San Francisco General Hospital, United States of America

## Abstract

**Background:**

Extensive evidence from observational studies suggests a role for genital herpes in the HIV epidemic. A number of herpes vaccines are under development and several trials of the efficacy of HSV-2 treatment with acyclovir in reducing HIV acquisition, transmission, and disease progression have just reported their results or will report their results in the next year. The potential impact of these interventions requires a quantitative assessment of the magnitude of the synergy between HIV and HSV-2 at the population level.

**Methods and Findings:**

A deterministic compartmental model of HIV and HSV-2 dynamics and interactions was constructed. The nature of the epidemiologic synergy was explored qualitatively and quantitatively and compared to other sexually transmitted infections (STIs). The results suggest a more substantial role for HSV-2 in fueling HIV spread in sub-Saharan Africa than other STIs. We estimate that in settings of high HSV-2 prevalence, such as Kisumu, Kenya, more than a quarter of incident HIV infections may have been attributed directly to HSV-2. HSV-2 has also contributed considerably to the onward transmission of HIV by increasing the pool of HIV positive persons in the population and may explain one-third of the differential HIV prevalence among the cities of the Four City study. Conversely, we estimate that HIV had only a small net impact on HSV-2 prevalence.

**Conclusions:**

HSV-2 role as a biological cofactor in HIV acquisition and transmission may have contributed substantially to HIV particularly by facilitating HIV spread among the low-risk population with stable long-term sexual partnerships. This finding suggests that prevention of HSV-2 infection through a prophylactic vaccine may be an effective intervention both in nascent epidemics with high HIV incidence in the high risk groups, and in established epidemics where a large portion of HIV transmission occurs in stable partnerships.

## Introduction

Human immunodeficiency virus (HIV) infection continues to be among the leading causes of global morbidity and mortality, especially in Africa. An estimated 40 million people are HIV infected with an annual mortality of three million [1]. The rapid spread of HIV as a sexually transmitted infection is exceeded by that of herpes simplex virus type 2 (HSV-2) [2], [3], [4]. The trajectories of HIV and HSV-2 incidence and prevalence contrast with the decline of bacterial sexually transmitted infections (STIs) in sub-Saharan Africa [5], [6], [7], [8], [9]. Indeed, the prevalence of HSV-2 has already reached high seroprevalence, of up to 90% in HIV-positive persons, and HSV-2 is now the leading cause of genital ulcer disease (GUD) in both developing and developed countries [2], [4], [10], [11].

The chronic nature of HSV-2 infection with frequent and mostly unrecognized reactivations [12], [13], and its relatively large transmission probability per coital act [14], [15], leads HSV-2 to having a different epidemiology among STIs. As opposed to bacterial infections such as gonorrhoea or syphilis, the threshold for sustainable transmission of HSV-2 at the population level is comparatively very low (Table 1). While bacterial STIs tend to be concentrated in high risk groups [16], HSV-2 transmission is sustainable in the general population, and therefore its prevalence can reach very high levels such as those observed in diverse regions around the globe [4], [17]. Consequently, the epidemiological overlap between HSV-2 and HIV is substantially larger than that of any other bacterial STI with HIV. This is supported by the epidemiological data that indicates that globally HIV-1 and HSV-2 have overlapping prevalence patterns [17], [18], [19], [20], [21].

**Table 1:** Threshold for sustainable transmission and transmission probability per partnership for key sexually transmitted infections*.

Agent	Duration of infectiousness in years	Transmission probability per partnership			Effective mean rate of new sexual partner acquisition per year for sustainable transmission
		One-year partnership	Ten-year partnership	Twenty-year partnership	
Neisseria gonorrhoeae (gonorrhea) [16], [93]	0.5	0.50	0.50	0.50	4
Chlamydia trachomatis (chlamydia) [16], [119]	1.25	0.20	0.20	0.20	4
Treponema pallidum (syphilis) [16], [94]	0.5	0.30	0.30	0.30	7
Haemophilus ducreyi (chancroid) [16], [120]	0.08	0.80	0.80	0.80	15
Herpes simplex sirus type 2 (HSV-2)	chronic reactivations for lifetime	0.20	0.89	0.99	0.4† ‡
Human immunodeficiency virus type 1 (HIV-1)	10	0.16	0.83	0.83	1.4^§^

* Appendix S1 includes the formulae used for our calculations for HIV and HSV-2. The values for the rest of the STIs are extracted from the cited references.

† Note that although HSV-2 transmission probability per partnership is not much larger than that of HIV, HSV-2 infectious spread is much more invasive since the absence of disease mortality allows HSV-2 infected persons to spread the infection in more sexual partnerships over their lifetime.

‡ The minimum effective new sexual partner acquisition rate is calculated assuming mean representative partnership duration of six months (Protocol S2).

The epidemiological overlap between the two viruses, and the nature of HSV-2 infection as a leading cause of clinical and sub-clinical genital ulceration and mucosal disruption, have focused a role for HSV-2 in the elevation of the HIV pandemic since the late eighties [22]. Two systematic reviews of 18 longitudinal studies showed that HSV-2 seropositivity has a relative risk of 2 or higher for HIV acquisition, after controlling for sexual behavior [23], [24]. These epidemiologic observations have been corroborated by evidence of biological plausibility. CD4+ lymphocytes, HIV target cells, have been detected in herpetic lesions [25], [26], which could increase HIV susceptibility during sexual exposure.

Further, the evidence that HSV-2 increases HIV transcription *in vitro* [27], [28], [29], [30], [31] potentially explains the high levels of HIV-1 RNA seen in herpetic lesions [32] and in plasma in dually infected patients [33], [34], [35], and supports higher HIV infectivity in dually infected subjects [36]. This has been corroborated by epidemiologic studies that suggested a relative risk of two to five fold of transmitting HIV from persons co-infected with HSV-2 compared to persons who are HSV-2 seronegative [37], [38]. In addition, HIV infection has been shown to substantially increase mucosal HSV-2 shedding [39], [40], [41], [42], [43], [44], [45]. These observations led to several proof-of-concept placebo-controlled trials to assess the effect of HSV-2 suppression on HIV infectiousness. The trials demonstrated significant reductions of up to 0.5 log_10_ in plasma viral load and up to 0.3 log_10_ in genital HIV load among HIV-HSV-2 coinfected persons during HSV-2 suppression [46], [47], [48], [49], [50], [51].

This body of knowledge suggests an epidemiologic synergy between the two diseases at the population level [52]. Some features of this interplay have been explored using mathematical models [5], [6], [53], [54]. Furthermore, population ecological studies have examined this conjecture. Most notably the cross-sectional population-based Four City study examined the differential spread of HIV in four sub-Saharan Africa cities in the late nineties [11]. It found that differences in reported sexual behavior patterns could not explain the disparity in HIV spread [55], [56], [57], and concluded that biological mechanisms, primarily male circumcision and HSV-2 infection, may explain the stark differences in HIV prevalence across the four cities [58].

The epidemiologic synergy of the two infections may be a factor in explaining the lack of effect of two large STI intervention trials in reducing HIV incidence [59], [60], [61]. The interventions involved primarily syndromic management or mass treatment of bacterial STIs, and did not incorporate HSV-2 treatment even though HSV-2 was far more prevalent than all other bacterial STIs combined [62]. For example in Rakai, genital herpes was identified to be the cause of almost half of all genital ulcers in the mid nineties during the STI treatment trial [63].

Thus, HSV-2 could have a major role in fueling HIV spread and, if HSV-2 could either be prevented or suppressed, potentially significant numbers of HIV infections could be averted. At present no vaccine to prevent HSV-2 acquisition or reactivation has been developed while the antivirals acyclovir, valaciclovir and famciclovir have been shown to be safe and effective in reducing HSV-2 shedding frequency and duration of genital ulcer disease [64], [65], [66]. Several trials have been conducted or currently under progress to measure the effect of acyclovir suppressive therapy in reducing HIV acquisition among HIV seronegatives, reducing HIV transmission from HIV and HSV-2 dually infected persons, and reducing HIV disease progression [67]. Two recent trials reported no efficacy of HSV-2 suppressive therapy on reducing HIV acquisition [68], [69]; an unexpected outcome considering the extensive and consistent evidence for epidemiologic synergy between the two infections. The mechanisms behind this failure are as yet unclear and may involve compliance to daily medication as well as the inability of the medication or regimen to suppress all types of subclinical reactivation. For example, the immune response to the HSV-2 virus in the genital tract may have not been reduced with therapy despite the reduction in ulcerations. Immune response to the HSV-2 virus in the genital tract, as indicated by increased density of CCR5+ CD4 cells and immature dendritic cells [70] and localization of HSV-specific CD8 cells at genital HSV-2 reactions [71], may have not been reduced with therapy despite the partial reduction in ulcerations (37% in overall GUD reduction and 64% reduction in HSV-2 DNA in ulcers [68]).

Furthermore, the relative effect of HSV-2 on HIV acquisition versus HIV transmission is not yet known and it is possible that most of the effect could be on enhancing HIV infectiousness rather than acquisition. The Partners in Prevention trial, in which the HIV/HSV-2 co-infected partner is randomized to HSV-2 suppression or placebo, will shed light on the impact on infectiousness [67]. Already several proof-of-concept placebo-controlled trials to assess the effect of HSV-2 suppression on HIV viral load, the principal predictor of HIV transmission probability per coital act [36], have demonstrated reductions of up to 0.5 log_10_ in plasma viral load and up to 0.3 log_10_ in genital HIV load among HIV-HSV-2 coinfected persons [46], [47], [48], [49], [50], [51]. Besides reducing HIV infectiousness, these reductions in viral load may imply slower disease progression to AIDS and death [67].

Freeman *et al.* have recently presented an analysis of the role of HSV-2 in HIV infectious spread based on the role of HSV-2 in causing GUD [72]. However, numerous studies in the last few years have shown that at least 75%, of all mucosal ulcerations due to HSV-2 in males and females are not clinically apparent [73], [74], [75]. “Subclinical” ulcerations that are not in noticeable anatomic areas, such as in the cervix or rectum, or are too small to be apparent on the vulva or penis, are much more common than clinical manifestations of herpes reactivations. In a recent study in which samples for mucosal detection of HSV-2 were taken 4 times daily, over 50% of HSV-2 ulcerations lasted less than 12 hours and only 15% of all episodes of HSV-2 shedding were associated with noticeable lesions [76]. Notably, no differences in HSV-2 shedding and reactivation have been observed among HSV-2 seropositive persons who do not report a history of GUD compared to those with a history of GUD [12]. Natural history studies have also shown small alterations in reactivation patterns of individuals over 10 years, and drug treatment trials involving persons treated for 6–10 years have shown the virus persists [77]. This paper provides our analysis of the interaction between HIV and HSV-2 based on the biological observation that subclinical ulcerations and reactivations play the leading role, as opposed to GUD, in driving the epidemiological synergy between HIV and HSV-2 seen in observational studies [23], [24]. Our approach to this analysis is further supported by the numerous clinical and laboratory based studies that have shown the upregulation of HIV during subclinical HSV-2 infection [27], [28], [29], [30], [31], [32], [33], [34], [35], as well as the recent evidence that subclinical HSV-2 infection is associated with the detection of higher copies of mucosal HIV RNA in the trials that demonstrated significant reductions in HIV plasma viral load and genital HIV shedding during HSV-2 suppression irrespective of clinical symptoms [46], [47], [48], [49].

To better assess the impact of HSV-2 on HIV epidemiology, we estimate the magnitude of the population-level epidemiologic synergy between the two viruses implied by the interaction demonstrated at the individual level. We address this question by synthesizing the biological and epidemiological observations into a mathematical model from which quantitative predictions can establish the magnitude of the synergy. Although the biological interaction between HIV and HSV-2 has been a subject of investigation by numerous studies, the parameters of some components of the interaction are not yet satisfactorily determined. The fact that both viruses share the same route of transmission, through sexual contact, makes it challenging to disentangle biological from behavioral mechanisms as confounding by sexual behavior [52], [78], [79], [80] may be one explanation for the substantial variability in study outcomes [23], [24]. The diversity of study designs and the heterogeneity in individual HSV-2 reactivation rates [12] further complicate estimations of some parameters [24]. With these considerations in mind, we investigate the synergy under two sets of assumptions for the interaction parameters. We also explore the size of the synergy in different settings and examine the differences between the role of HSV-2 in enhancing HIV acquisition versus its role in enhancing HIV infectivity.

## Methods

### Model structure

A deterministic compartmental model was constructed to describe HIV and HSV-2 transmission and acquisition dynamics and their interaction. The model consists of thirty-two coupled nonlinear ordinary differential equations that stratify the population into compartments according to HIV infection status and stage, HSV-2 infection status and stage, and the sexual risk activity class. HIV progression is divided into three stages of acute, chronic, and advanced, while HSV-2 infection is depicted by the three stages of primary infection, latent infection, and reactivation. Dual infection is characterized by nine stages according to each of HIV and HSV-2 stages. The population is divided into two sexual risk behavior classes of low-risk population (“general population”) and high risk group (“core group”). The mixing between the sexual activity classes has assortative (choosing partners only from within their risk group) and proportionate (choosing partners with no preferential bias based on the kind of risk group) components.

Our model calculates the size of the synergy between the two infections where the synergy is defined as the combined effect on HIV epidemiology of the presence of enhanced risk of HIV acquisition in HSV-2 seropositive subjects, enhanced HIV infectivity in dually infected persons, and enhanced HSV-2 shedding in dually infected persons. The structure of the model along with all parameter definitions are delineated in Protocol S1.

### Key assumptions and parameter values

The parameter values of the model are chosen according to the best available empirical evidence of the biology, epidemiology, and interaction of the two infections. In particular, this is the first modeling assessment where the recently established detailed empirical data about the pattern of HSV-2 reactivation in its clinical and subclinical form has played a central role in the model. Table 2 lists the key assumptions of the model. The per-exposure cofactor provides the multiplicative impact of a given risk on HIV transmission probability per coital act. The susceptibility enhancement per-exposure cofactor of *PEC_Acq_* = 4 and *PEC_Acq_* = 9 during HSV-2 primary infection and reactivations (i.e. HSV-2 shedding), irrespective of whether HSV-2 infection is manifested clinically or subclinically, are derived in Protocol S2 from the overall risk ratios (*RR*) of HIV acquisition in HSV-2 seropositive persons as estimated in two meta-analyses at *RR* values of 2.1 [24] and 2.9 [23], respectively. We assume an infectivity enhancement per-exposure cofactor of *PEC_Trans_* = 3 in dually infected persons during HSV-2 primary infection and reactivations irrespective of clinical symptoms as a representative value of the combined effect of HSV-2 induced increase in HIV transcription [27], [28], [29], [30], [31], the measured heightened HIV plasma viral load in dually infected patients [33], [34], [35], [46], [47], [48] (and its implication in terms of HIV infectiousness [36]), and the presence of HIV-1 RNA in herpetic lesions [32]. Protocol S2 includes more discussion of the rationale for this value. We hypothesize that the enhanced susceptibility and infectivity do not occur in absence of HSV-2 shedding in view of the lack of a plausible biological mechanism during HSV-2 latency.

**Table 2:** The key assumptions of the HIV/HSV-2 interaction model.

Parameter	Value	References
HIV transmission probability per coital act per stage of HIV infection:		
Acute stage	0.0107	[88], [115] (Protocol S2)
Chronic stage	0.0008	[88], [115] (Protocol S2)
Advanced stage	0.0042	[88], [115] (Protocol S2)
Duration of each of HIV stages:		
Acute stage	2.5 months	[115]
Chronic stage	7.59 years	[83], [84] (Protocol S2)
Advanced stage	2.0 years	[115]
Susceptibility enhancement to HIV acquisition per-exposure cofactor during HSV-2 shedding (*PEC_Acq_*)	4.0	derived based on meta-analysis in [24] (Protocol S2)
		Figures 1, 3 (for the *PEC_Acq_* = 4 calculations), 4A, 4C, and 5
	9.0	derived based on meta-analysis in [23] (Protocol S2)
		Figures 2, 3 (for the *PEC_Acq_* = 9 calculations), 4B, and 4D
HIV infectivity enhancement per-exposure cofactor in dually infected subjects during HSV-2 shedding (*PEC_Trans_*)	3.0	representative assumption based on [33], [34], [35], [46], [47], [48] (Protocol S2)
HSV-2 shedding frequency among:		
HIV susceptible persons	14% of the time	[76]
Acute and chronic HIV persons	20% of the time	[17], [43], [76] (Protocol S2)
Advanced HIV persons	31% of the time	[17], [43], [76] (Protocol S2)
HSV-2 transmission probability per coital act		model fit informed by [14], [15] (Protocol S2)
	0.0116	Figures 1, 3 (for Kisumu at *PEC_Acq_* = 4), 4A, and 4C
	0.0144	Figures 2, 3 (for Kisumu at *PEC_Acq_* = 9), 4B, and 4D
	0.00343 (*PEC_Acq_* = 4) and 0.00407 (*PEC_Acq_* = 9)	Cotonou calculation model fit in Figure 3
	0.00632 (*PEC_Acq_* = 4) and 0.00757 (*PEC_Acq_* = 9)	Yaoundé calculation model fit in Figure 3
	0.0084 (*PEC_Acq_* = 4) and 0.01021 (*PEC_Acq_* = 9)	Ndola calculation model fit in Figure 3
Duration of the HSV-2 cycle of latency and reactivation	4 per year	[13]
Duration of HSV-2 stages:		
Primary infection	20.0 days	representative assumption informed by [13] (Protocol S2)
Latency between HSV-2 reactivations for HIV seronegative persons	78.5 days	derived (Protocol S2)
Latency between HSV-2 reactivations for HIV seropositive persons in acute or chronic stages	73.0 days	derived (Protocol S2)
Latency between HSV-2 reactivations for HIV seropositive persons in advanced stage	63.0 days	derived (Protocol S2)
Shedding during reactivation for HIV seronegative persons	12.8 days	derived (Protocol S2)
Shedding during reactivation for HIV seropositive persons in acute or chronic stages	18.3 days	derived (Protocol S2)
Shedding during reactivation for HIV seropositive persons in advanced stage	28.3 days	derived (Protocol S2)
Frequency of coital acts:		
Acute stage	10.6 per month	[115]
Chronic stage	11.0 per month	[115]
Advanced stage	7.1 per month	[115]
Fraction of the high risk group in the population	11.3%	derived based on behavioral measures in [55], [56], [57] (Protocol S2)
Number of sexually active people in the population at the start of the simulation	200,000	representative assumption based on [57], [121], [122], [123] (Protocol S2)
Number of people in the low risk group at the start of the simulation	177,400	derived (Protocol S2)
Number of people in the high risk group at the start of the simulation	22,600	derived (Protocol S2)
The effective new sexual partner acquisition rate:		representative assumptions based on model fits and informed by [55], [56], [57] (Protocol S2)
Low-risk population	0.406 partners per year	model fit in Figures 1, 3 (at *PEC_Acq_* = 4), 4A, and 4C
	0.371 partners per year	model fit in Figures 2, 3 (at *PEC_Acq_* = 9), 4B, and 4D
	0.401 partners per year	model fit in Figure 5
High risk group	26.000 partners per year	model fit in Figures 1, 3 (at *PEC_Acq_* = 4), 4A, and 4C
	21.000 partners per year	model fit in Figures 2, 3 (at *PEC_Acq_* = 9), 4B, and 4D
	30.000 partners per year	model fit in Figure 5
Degree of assortativeness	0.2	representative assumption based on model fit and informed by [55], [56], [57] (Protocol S2)
Duration of sexual partnerships:		
Among the low-risk population	36 months	representative assumption informed by [55], [56], [57] (Protocol S2)
Among the high risk population	1 months	representative assumption informed by [55], [56], [57] (Protocol S2)
Between the low-risk and high risk populations	6 months	representative assumption informed by [55], [56], [57] (Protocol S2)
Duration of the sexual lifespan	35 years	[124]

Although there are substantial variations in the pattern (and frequency) of HSV-2 reactivations [13], [76], the critical parameter here is the shedding frequency irrespective of whether the pattern is that of short but frequent reactivations or long but less frequent ones since by assumption the synergy acts whenever there is shedding and irrespective of the pattern of shedding. The model predictions were invariable by keeping the shedding frequency fixed, but varying the pattern of shedding.

Prospective partner and time to HSV-2 studies indicate a value for HSV-2 transmission probability per coital in the range of 0.0005 to 0.022 [14], [15]. We derived two rough estimates for this probability using the cohort data of Corey *et al.* [64] and the EXPLORE behavioral intervention study of men who have sex with men [81]. The calculations suggest a value of 0.01 which is in the middle of the range indicated by prospective partner and time to HSV-2 studies (Protocol S2). In absence of precise estimates, we use the HSV-2 transmission probability per coital act as one of the model fitting parameters, but constrain its value to be in the range suggested by the empirical data. The model fitting suggests a value in the neighborhood of 0.01 which is consistent with the derived rough estimates.

Due to insufficient evidence, we conservatively assume that dual infection does not increase HSV-2 transmission probability per coital act or HIV disease progression [82], [83], [84], [85], although HSV-2 seropositivity was reported to increase systemic HIV virus levels significantly during acute and chronic HIV infection [86]. We further assume that HIV seropositivity does not biologically enhance the susceptibility to HSV-2 infection. The behavioral parameters in the model are informed by the measurements of the Four City study [55], [56], [57]. Their exact values are obtained through the model fits which yield reasonable estimates for these parameters (Protocol S2 and Table 2). More details regarding the model parameters and their related analyses can be found in Protocol S2.

### Measures of epidemiologic synergy

We estimate the size of the epidemiologic synergy first using the population attributable fraction of HIV due to HSV-2 infection defined as
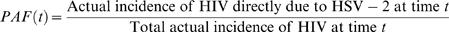
This definition expresses the direct role of HSV-2 in HIV incident cases at each time point *t* in the epidemic [87]. We define three *PAF* measures. First, *PAF_Sus_*(*t*) which measures the percentage of all incident HIV infections at time *t* that are directly caused by the enhanced susceptibility of HSV-2 seropositive persons to HIV acquisition [24]. Second, *PAF_Inf_*(*t*) which measures the percentage of all incident HIV infections at time *t* that are directly caused by the enhanced HIV infectivity in dually infected persons. Third, *PAF_Sus_*_+*Inf*_(*t*) which measures the percentage of all incident HIV infections at time *t* that are directly caused by HSV-2 whether due to enhanced susceptibility or infectivity.

The *PAF_Sus_*_+*Inf*_ is not simply a sum of *PAF_Sus_* and *PAF_Inf_* since there are incident HIV cases that can be attributed causally to both enhanced susceptibility and enhanced infectiousness. This occurs if an HSV-2 seropositive subject in sexual partnership with a dually infected person acquires HIV because HSV-2 increases concurrently the susceptibility to HIV and the infectivity of the HIV/HSV-2 coinfected partner. Therefore, *PAF_Sus_*_+*Inf*_≤*PAF_Sus_*+*PAF_Inf_* since some incident cases are counted in each of *PAF_Sus_* and *PAF_Inf_*, but counted only once in the *PAF_Sus_*_+*Inf*_.

Accordingly, the *PAF* estimates the fraction of incident HIV cases that are caused by the direct biological effects of HSV-2. But as opposed to non-infectious diseases, any HIV infection caused by HSV-2 can also generate onward transmission of secondary HIV infections regardless of HSV-2 as a cofactor. Such secondary infections are considered indirect effects of HSV-2 role in HIV epidemiology and are not counted in the *PAF* measures. However, these indirect effects affect HIV epidemiology and prevalence. Therefore we use HIV excess prevalence [88] as an additional measure of the synergy. HIV excess prevalence is defined as the difference in HIV prevalence in a scenario where there is a biological interaction between the two infections and a counter-factual scenario where there is no biological interaction between the two diseases. Analogously, we define HSV-2 excess prevalence which provides a measure of the impact of HIV on HSV-2 epidemiology.

### Uncertainty and sensitivity analyses of the key parameters and basic assumptions in the model

We conducted uncertainty and sensitivity analyses of the key parameters in the model, examined the size of the epidemiologic synergy as a function of susceptibility and infectivity enhancements, and discussed the impact of structural changes in the model such as explicit incorporation of other sexually transmitted infections in the model. The details of these analyses can be found in Protocol S3.

## Results

### Analysis

**Figure 1:**
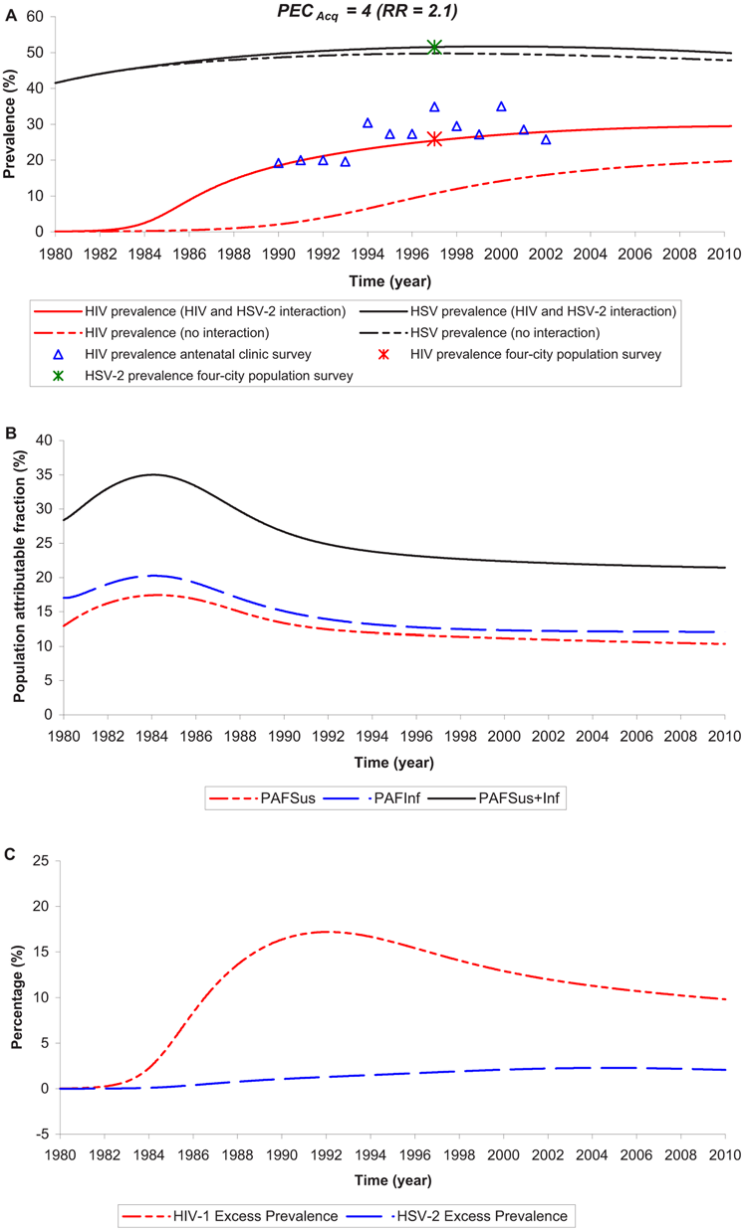
Time course of the HIV epidemic in Kisumu, Kenya. Here assuming a susceptibility enhancement per-exposure cofactor of *PEC_Acq_* = 4 (implying a *RR* = 2.1 [24]). The upper panel (A) shows HIV and HSV-2 prevalences in the presence of the two disease interaction compared to the prediction in the absence of interaction. The references for the data are discussed in Protocol S2. The middle and lower panels show the epidemiologic synergy measures of HIV and HSV-2 interaction. The middle panel (B) displays the fraction of all incident HIV infections that are directly caused by the enhanced susceptibility of HSV-2 seropositive persons to HIV acquisition (*PAF_Sus_*), the fraction of all incident HIV infections that are directly caused by the enhanced HIV infectivity in dually infected persons (*PAF_Inf_*), and the fraction of all incident HIV infections that are directly caused by HSV-2 biology whether due to enhanced susceptibility or enhanced infectivity (*PAF_Sus_*_+*Inf*_). The lower panel (C) shows HIV and HSV-2 excess prevalences.

We examine the impact of the interaction in Kisumu, Kenya, a setting with relatively high HIV and HSV-2 prevalences, as a representative locality to examine the epidemiologic synergy. Kisumu is also one of the four cities in the Four City study that assessed the differential prevalence of HIV in sub-Saharan Africa [89]. We first examine the impact under the assumption that *PEC_Acq_* = 4 (implicitly *RR* = 2.1 [24] (Protocol S2)). Figure 1A depicts the time course of the epidemics in terms of prevalences in the presence of the two disease interaction and compare it to the prevalences in a counter-factual scenario where no interaction is assumed. Figure 1B and 1C display the *PAF_Sus_*, *PAF_Inf_*, *PAF_Sus_*_+*Inf*_, and HIV and HSV-2 excess prevalences.

Roughly 25% of all incident HIV infections throughout the epidemic are attributed directly to HSV-2 (Figure 1B). Half of these infections were caused by the enhanced susceptibility of HSV-2 seropositives to HIV infection while the other half was caused by the enhanced HIV infectivity in dually infected patients. Although *PEC_Acq_* = 4>*PEC_Trans_* = 3, the effect of enhanced infectivity was slightly larger as dually infected persons have more frequent HSV-2 reactivations.

The HIV and HSV-2 excess prevalences in Figure 1C illustrate how HSV-2 has played a major role in fuelling HIV spread directly and indirectly particularly in the early stage of the epidemic though HIV has limited impact on HSV-2 prevalence throughout the HIV epidemic. This confirms earlier findings that HSV-2 [53] and other STIs [6], [7] may have accelerated the evolution of the HIV epidemic. However, though the direct role of HSV-2 in causing 25% of HIV infections remained rather stable throughout the epidemic, the indirect effect in terms of secondary HIV infections and onward HIV transmission declined steadily with time as the number of persons susceptible to HIV diminished due to the rising HIV prevalence.

**Figure 2:**
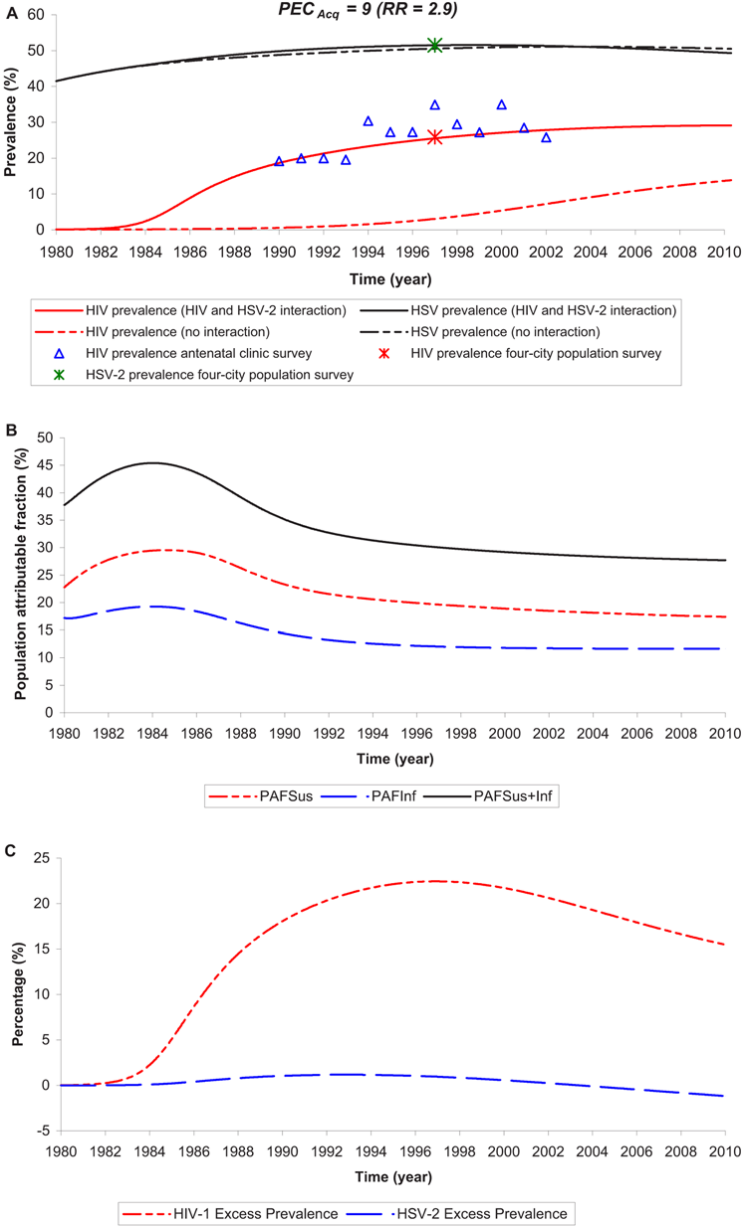
Time course of the HIV epidemic in Kisumu, Kenya. As in Figure 1, but now assuming *PEC_Acq_* = 9 (implying *RR* = 2.9 [23]).

Figure 2 displays the anticipated impact of the interaction if the *PEC_Acq_* = 9 (implicitly *RR* = 2.9 [23] (Protocol S2)). Here roughly 35% of all incident HIV infections throughout the epidemic are attributed directly to HSV-2. Roughly two-third of these infections were caused by the enhanced susceptibility to HIV and one-third was caused by the enhanced HIV infectivity.

**Figure 3:**
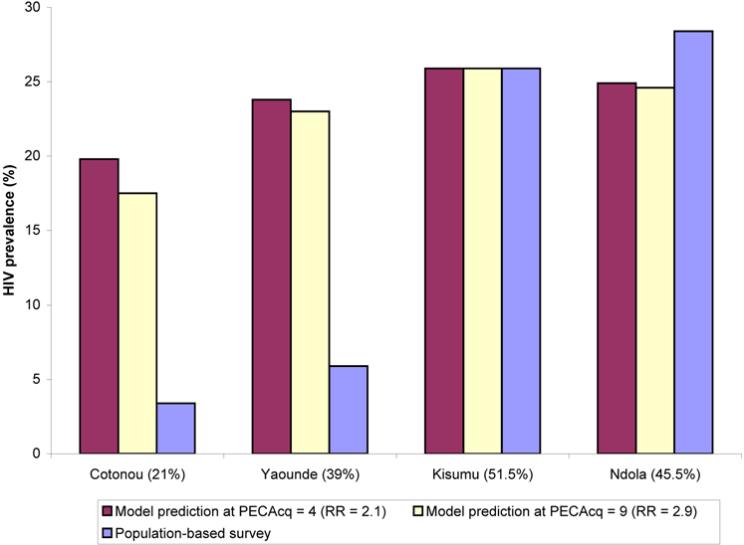
HSV-2 and the findings of the Four City study [58]. HIV prevalence in 1997 in the four cities as predicted by the model for the scenarios of *PEC_Acq_* = 4 and *PEC_Acq_* = 9 compared to HIV prevalence as measured in the survey [89], versus HSV-2 prevalence in the four cities [11]. The calculations were done using the same sexual behavior parameters as those in Kisumu. The figure estimates the magnitude of HSV-2 contribution to the differential HIV prevalence across the four cities.

The above results indicate that at the time of the Four City study (1997–1998), the interaction has contributed 14% in excess HIV prevalence if *PEC_Acq_* = 4 (Figure 1C) and 22% if *PEC_Acq_* = 9 (Figure 2C). To assess the role of HSV-2 in contributing to the differences in HIV prevalence between the cities of the Four City study [58], we calculated HIV prevalence in 1997 using all other biological and behavioral parameters fixed to those in Kisumu, but imposing HSV-2 prevalence levels as those found in the four cities of the survey [11] (Figure 3). Notably the Four City study found sexual-risk behavior measures to be similar overall across the four cities [55], [56], [57], [58] suggesting that our results may be generalizable to other settings that share Kisumu's HIV and HSV-2 prevalence levels. We performed the calculations for the scenarios of *PEC_Acq_* = 4 and *PEC_Acq_* = 9 by varying HSV-2 transmission probability per coital act to fit HSV-2 prevalence in the four cities. Figure 3 shows HIV prevalence as predicted by the model compared to the prevalence as measured in the survey versus the four HSV-2 prevalence levels. The calculations signify that HSV-2 indeed explains part of the gap in HIV prevalence between the low and high prevalence cities. If HSV-2 prevalence in Kisumu was at 21% as in Cotonou, Benin, then HIV excess prevalence would have been 6.1% (if *PEC_Acq_* = 4) or 8.4% (if *PEC_Acq_* = 9). These values suggest that the epidemiologic synergy between HIV and HSV-2 accounts for about a third of the gap in HIV prevalence (roughly 6–9% out of 20%) in Kisumu compared to Cotonou. This finding highlights how HSV-2, in addition to other biological cofactors such as male circumcision [90], can fuel and maintain a substantial differential prevalence of HIV across diverse geographic areas that have similar sexual risk behavior patterns.

**Figure 4:**
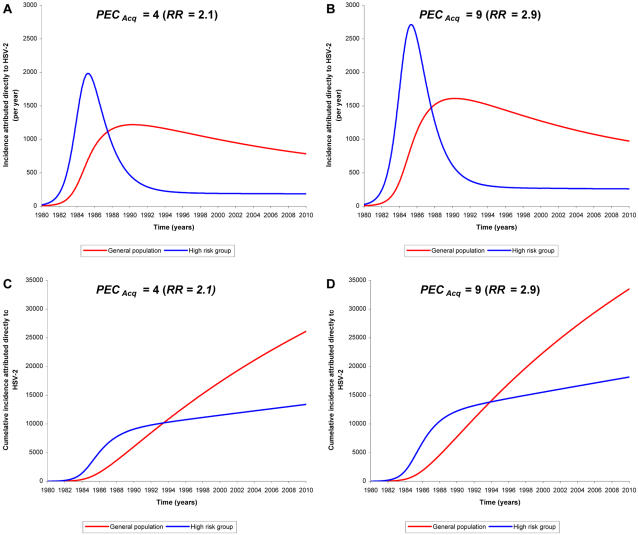
HSV-2 role in different risk groups. Role of HSV-2 in fueling HIV spread per sexual activity group in the calculations of Figure 1 (A and C) and Figure 2 (B and D). The upper panels A and B show the time course of HIV incidence per year attributed directly to HSV-2 in the low-risk population and in the high risk group. The lower panels C and D display the cumulative incidence of HIV infections since 1980 (actual number of infections) attributed directly to HSV-2 in the low-risk population and in the high risk group.

Figure 4A and 4B show the incidence of HIV infections attributed directly to HSV-2 per risk group in the Kisumu calculations of Figure 1 and 2 respectively, and Figure 4C and 4D display the cumulative incidence (actual number of HIV infections) per risk group since 1980 for the same calculations. The results illustrate the key role of HSV-2 in the dynamics of HIV transmission, irrespective of the value of *PEC_Acq_*, and its large impact in the low-risk population with stable and long-term partnerships. Initially, HSV-2 facilitated HIV transmission mainly in the high risk groups, but this effect was short-lived as the high risk population was quickly infected with HIV. By the late 1980s, the number of incident infections attributed to HSV-2 in the high risk population decreased substantially due to saturation in the high-risk population, but increased among the low-risk population peaking in the early 1990s. As opposed to the high risk group, the epidemic phase of HIV was slow to decline and the cumulative number of new infections attributed to HSV-2 continues to increase. By 1994 more than 50% of the cumulative number of HIV infections attributed directly to HSV-2 were estimated to have occurred in the low-risk population (Figure 4C and 4D). The role of HSV-2 in the later stages of the HIV epidemic, as opposed to bacterial STIs, has been also reported by Orroth *et al.* [54] and Freeman *et al.* [72]

**Figure 5:**
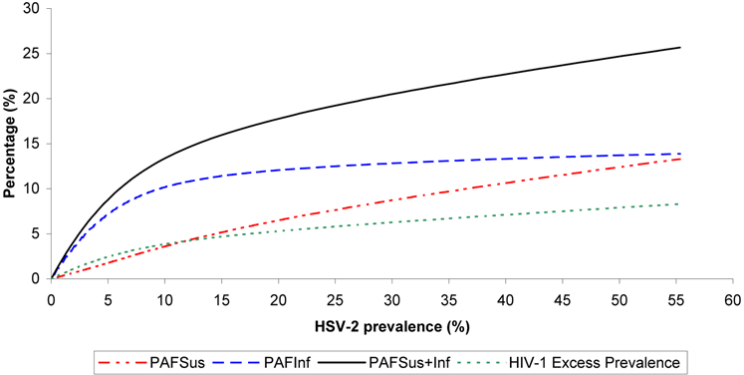
Epidemiologic synergy between HIV and HSV-2. Measures of the impact on HIV of the epidemiologic synergy between the two infections in diverse HSV-2 settings. The *PAF_Sus_*, *PAF_Inf_*, *PAF_Sus_*_+*Inf*_, and HIV excess prevalence as a function of HSV-2 prevalence. The HIV prevalence in absence of interaction is at 20%.

In addition to examining the role of HSV-2 in a specific epidemic, we examined the impact of HSV-2 in diverse settings assuming *PEC_Acq_* = 4. Figure 5 displays the epidemiologic synergy measures assuming 0–60% HSV-2 prevalence, where this range reflects the global variability of HSV-2 prevalence among the general population [2], [3], [4]. The measures are calculated at the endemic equilibrium to disentangle interaction-strength effects from temporal effects. As indicated in the figure, the level of HSV-2 prevalence dictates the magnitude of the impact on HIV. When HSV-2 seropositivity is high, up to 25% of incident HIV infections are etiologically attributed to HSV-2. The impact of enhanced susceptibility to HIV increases moderately, but steadily, with HSV-2 prevalence. However, the impact of HSV-2 of enhanced HIV infectivity increases rapidly up to HSV-2 prevalence of 10% and then increases slowly. The rapid increase in *PAF_Inf_* reflects HIV spread in the high risk group, here comprising 11% of the population, while the slow growth represents the spread in the low risk group following saturation in the high risk group. This result underscores how dually infected persons in the high risk group are essentially a group of HIV “super-spreaders” since the effect of their higher infectivity is further amplified by their behavior as well as their sexual networks and their partners' high risk behavior. High risk groups in areas where HSV-2 prevalence is low but rising should be a primary target of HIV and HSV-2 interventions.

The impact of genital herpes on HIV epidemiology can be analytically understood in terms of the impact of the interaction on the HIV basic reproductive number (*R*_0_); the number of secondary infections that an index case would generate upon entrance into an infection-free population [91]. In order for an infection to spread, *R*_0_ needs to be larger than one. If *R*_0_<1, the number of secondary infections each index case produces is on average less than one and the infection cannot sustain itself and dies out. If *R*_0_>1, but still close to *R*_0_ = 1, the prevalence increases rapidly with each incremental increase in *R*_0_. If *R*_0_ is much larger than one, the prevalence becomes insensitive to any further increases in *R*_0_ due to susceptible depletion. Hence, the most consequential change in the biology of an infectious pathogen is when it makes the transition from a non-sustainable infection to a rapidly spreading infection when it crosses the threshold at *R*_0_ = 1.

Since sexual risk behavior is characterized by substantial heterogeneity, each risk group in the population has its own *R*_0_. An STI can be sustainable and endemic in some risk groups (such as sex workers) while being below the sustainability threshold in other groups. Indeed, this is the case for bacterial STIs such as gonorrhea and syphilis [16], [92], [93]. These infections are sustainable only if the effective new sexual partner acquisition rate (ρ), is larger than an estimated 4 (gonorrhea) [16], [93] and 7 (syphilis) [16], [94] partners per year (Table 1). Though these infections can still spread in the lower risk groups, the chain of transmission is not sustainable.

The variability in the infectious spread of STIs per risk group leads to the concept of group of sustainable transmission (GST) [16], [92], [93]. The GST for a specific STI is the population where the infection's chain of transmission is sustainable (*R*_0_>1). It bears notice that the GST is sometimes labeled as the core group of transmitters (CGT) [16], [92]. We adopted a different terminology here to avoid confusion with the concept of core group defined as a small subgroup of the population with extremely higher risk behavior than the rest of the population [95].

We calculated, using *R*_0_ and the equations described in Appendix S1, the minimal value of ρ that is needed to sustain each of HSV-2 and HIV infections in a uniform risk population in absence of synergy between the two infections. We found that HSV-2 is sustainable in any population with ρ>0.4 partners per year, while HIV needs at least a ρ = 1.4 partners per year for sustainability. Here and for all calculations of the threshold value of ρ we assumed an average representative partnership duration of 6 months (Protocol S2). Hence, HSV-2 has a much larger GST than HIV or any of the bacterial STIs (Table 1), and its GST may encompass the majority of the population in sub-Saharan Africa considering its observed prevalence [2], [3], [4] and reported sexual behavior measures [55], [56], [57], [89]. Despite the larger transmission probability per coital act of many bacterial STIs, the chronic nature of HSV-2 infection, compared to the short infectious period of bacterial STIs, leads to HSV-2 transmission probability per partnership growing steadily with the duration of partnership (Table 1 and Appendix S1).

**Figure 6:**
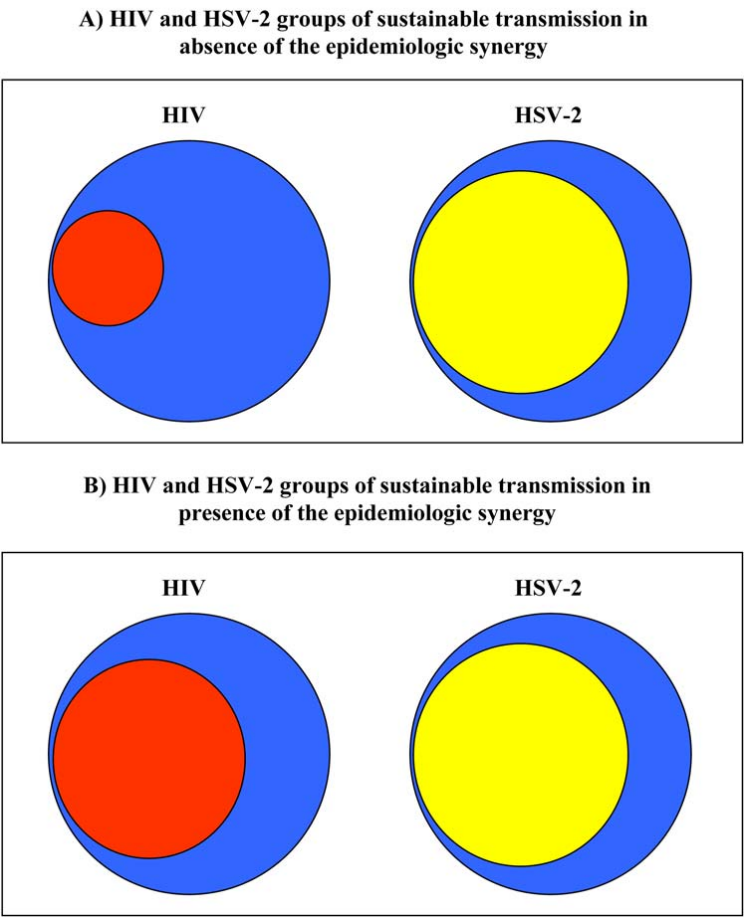
Schematic diagram of the role of HSV-2 in HIV epidemiology. The upper panel (A) shows the HIV and HSV-2 groups of sustainable transmission (GST) in absence of interaction between the two diseases while the lower panel (B) shows the groups of sustainable transmission in presence of the epidemiological synergy. The high HSV-2 prevalence in sub-Saharan Africa has facilitated a substantial increase in the size of the HIV group of sustainable transmission to encompass a large fraction of the low-risk population.

Although the HIV GST encompasses only a fraction of the population, the biological interaction between HIV and HSV-2 changes the dynamics considerably. The HSV-2 seropositive population with a ρ in the range of 1.1 to 1.4 per year if *PEC_Acq_* = 4 (and in the range of 0.9 to 1.4 if *PEC_Acq_* = 9), becomes effectively part of the HIV GST. These values were calculated numerically by finding the threshold value of ρ at which HIV prevalence at the endemic equilibrium becomes non-vanishing in a uniform risk population [96] . Hence, the synergy between HSV-2 and HIV allowed HIV to reach a substantially larger fraction of the general population than was possible without the biological interaction (Figure 6).

## Discussion

Our findings suggest a major role for HSV-2 in fueling HIV transmission and acquisition in Africa, where HSV-2 may account for 25% to 35% of incident HIV infections. The contribution of HSV-2 to HIV incidence is largely due to the role of HSV-2 in increasing the fraction of the population at risk of HIV acquisition and once co-infected, results in them becoming more infectious for HIV, and increasing the pool of HIV infected persons in the general population.

The results of the Four City study [58] corroborate our findings. In the two West African cities with lower HSV-2 prevalence, HIV remained concentrated in higher risk groups whereas in the cities with high HSV-2 prevalence, HIV transmission was observed in a large fraction of the population. Here, HSV-2 was a trigger for the explosive expansion of HIV. Lack of male circumcision may have impacted HIV epidemiology similarly [90], [97]. The role of HSV-2 in the HIV epidemic is in sharp contrast to the role of bacterial STIs. Bacterial STIs are concentrated in the high risk groups and principally served as a co-factor for increasing HIV susceptibility and infectiousness in the high-risk population, but their role diminished as HIV prevalence increased in the low-risk population [6], [7], [54], [98], [99], [100]. In contrast, HSV-2 has maintained its contribution to the epidemic as HIV moved to the low-risk population.

Our results indicate that genital herpes may explain as a much as a third of the gap in HIV prevalence between the four cities of the Four City study [11], [58]. Nevertheless, HSV-2 alone cannot explain the differential prevalence; and other factors, such as lack of male circumcision, likely play a role. Indeed, the Four City study [58], [97], numerous observational studies [101], a modeling study [90], and the striking magnitude of protection against HIV infection reported in three randomized trials of male circumcision [102], [103], [104], suggest also a substantial role for the lack of male circumcision in explaining the differences in HIV prevalence.

The evidence to date does not determine whether there is a biological interaction between HSV-2 and male circumcision. Since circumcision protection is constrained to the forehead of the penis while HSV-2 reactivation in the sacral ganglia affects a much larger surface area of the male genitalia [102], [105], the interaction between the two biological cofactors may be limited. A recent meta-analysis suggests no significant circumcision protection against HSV-2, despite protection against other ulcerative STIs [105]. Two of the male circumcision trials found no efficacy for male circumcision in reducing HSV-2 seroincidence [102], [103], [106], Bertran Auvert, private communication. Nevertheless, a reduction of GUD has been observed in two of the circumcision trials [103], [104], [106] suggesting the possibility of a protective effect against symptomatic HSV-2 reactivations. Noteworthy is that while circumcision offers protection for men, most of the HSV-2 impact on HIV is among women who are generally twice as likely to be HSV-2 seropositive [11].

We found that the excess prevalence of HSV-2 due to HIV is much smaller than that of HIV due to HSV-2 (Figures 1C and 2C). The impact of HIV on HSV-2 is mediated by the increased HSV-2 shedding in dually infected patients which is counterbalanced by AIDS mortality as HSV-2 positive persons are more likely to acquire HIV and die from AIDS. The impact of increased shedding is generally larger than that of AIDS mortality. The stability of HSV-2 prevalence throughout the HIV epidemic has been also seen in earlier modeling work on the role of STIs [5], [6], [107], and is consistent with the similar HSV-2 prevalence levels in Rakai and Mwanza despite the disparity in the severity of the two HIV epidemics [62]. Furthermore, the similar HSV-2 prevalence levels observed in a cohort in Uganda in the early 1990s [108] and in the Four City study in Kisumu in the late 1990s [11], suggest also that HSV-2 prevalence may have saturated at about 50% as a population average with little impact of HIV. The rapid HSV-2 epidemic expansion in Africa, which appears to have started in the first half of the twentieth century [109], seems to have reached its peak and saturated prior to the HIV epidemic in some parts of sub-Saharan Africa.

Since at least 75% of all mucosal ulcerations due to HSV-2 are not clinically apparent [73], [74], [75], the role of GUD due to HSV-2 in our model accounts for only a minor component, of no more than 25%, of the total impact of HSV-2 on HIV in contrast to the assessment in Freeman *et al.* [100]. The empirical evidence about HSV-2 natural history and its high frequency of subclinical reactivations is at odds with assuming such a large *PAF* due to GUD. In Rakai, the *PAF* due to GUD of all etiologies was estimated to be only about 20% including the effects on both HIV acquisition and transmission [61], which is consistent with our estimates. The high *PAF*s in Freeman *et al.* model, even for single GUD etiologies such as chancroid, are a consequence of the assumed large cofactor effects with GUD (for example a 25-fold increased per-contact transmission in the presence of chancroid) and the assumed high prevalence of GUD (such as almost 4% prevalence for chancroid in the early phase of the HIV epidemic). In our view these assumptions exaggerate the role of GUD in the HIV dynamics and are not supported by biological evidence [110].

Our approach for a larger role of HSV-2 subclinical ulcerations compared to GUD suggests that syndromic or episodic management of HSV-2 ulcers would have limited impact on reducing HIV incidence. However if HSV-2 suppressive therapy can effectively reduce either HIV acquisition or transmission, such therapy offers a potential tool to influence the HIV epidemic. Our analysis suggests that an effective therapeutic or prophylactic HSV-2 vaccine could also reduce HIV acquisition and transmission. The recent results of HSV-2 syndromic and suppressive therapy trials illustrate these issues. Syndromic therapy with acyclovir had limited impact on HIV plasma viral load and HIV genital shedding [111], [112], whereas suppressive therapy resulted in substantial reductions in both plasma and genital HIV shedding irrespective of the presence of clinical genital herpes symptoms [46], [47], [48], [49].

Limitations of this model include the possibility of sexual risk behavior confounding, in the measured relative risk ratios, affecting our quantitative predictions of the size of the epidemiologic synergy. The observed high risk of HSV-2 acquisition in HIV seropositive subjects relative to HIV seronegative persons [108], [113], despite the limited evidence for a compelling biological mechanism apart from immunosuppression due to HIV, is an indicator of the possibility of such confounding. There are also several methodological problems in the derivation and interpretation of HIV and STI biological cofactors, and measurements of the enhanced acquisition among susceptible partners can be inflated by exposure to the enhanced infectivity of their partners if the STI was present in both partners [114]. However, we minimized these limitations by the conservative assumptions we employed in our derivations of the per-exposure cofactors. We derived the *PEC_Acq_* from the *RR* assuming that the *RR* was measured in partnerships where both partners are HSV-2 seropositive (Protocol S2). We also found that the *PEC_Acq_* derived using the *RR* to be consistent with that derived using the Rakai data of HIV transmission probabilities (Protocol S2), where the role of confounding is likely minimal due to the study design of monogamous partnerships and confirmation of within partnership transmission [17], [37], [115]. Our predictions for the *PAF_Sus_*, and even for the *PAF_Sus_*_+*Inf*_, are still substantially smaller than those derived in empirical studies [24], [116], [117] using the classical Levin formula [118].

In conclusion, the prominent effect of HSV-2 in fueling the HIV epidemic in sub-Saharan Africa is related to 1) the biology of HSV-2 as a chronic infection and a leading cause of clinical and sub-clinical genital mucosal disruption, 2) the high HSV-2 prevalence, 3) enhanced HIV acquisition in HSV-2 seropositive persons, and 4) enhanced HIV infectivity in dually infected subjects. Our model projects that HSV-2 has played a leading role in fueling HIV spread in sub-Saharan Africa, and may be responsible for one-third of the differential HIV prevalence in the Four City study. Our current tools for reducing the impact of HSV-2, such as use of HSV-specific antivirals like acyclovir to suppress HSV-2 reactivation, may not be effective enough to reduce the effect of HSV-2 on increasing HIV susceptibility, which contributes to the HSV-HIV synergy. However, an HSV-2 vaccine may be an effective intervention to prevent the interaction of the two infections by removing HSV-2 as a risk factor for HIV acquisition and transmission. Additionally, ongoing trials will soon inform us of the potential role of antiviral suppression of HSV-2 to reduce HIV transmission and disease progression, which could still have an important impact in interrupting the synergy between these two viruses.

## Supporting Information

Appendix S1 Supporting mathematical equations
(0.06 MB DOC)

Protocol S1 HIV and HSV-2 interaction model description.
(0.06 MB PDF)

Protocol S2 Biological and behavioral parameters.
(0.11 MB PDF)

Protocol S3 Uncertainty and sensitivity analyses of the key parameters in the model.
(0.57 MB DOC)
