# Supplementary material for: Genital Herpes Has Played a More Important Role than Any Other Sexually Transmitted Infection in Driving HIV Prevalence in Africa
Source: PLoS One. 2008 May 21;3(5):e2230. doi: 10.1371/journal.pone.0002230 (PMC2377333; doi:10.1371/journal.pone.0002230)
Supplement: Appendix S1 — Supporting mathematical equations (0.06 MB DOC) [file pone.0002230.s001.doc]

**Appendix S1**

***Transmission probability per partnership***

The transmission probability per partnership is assumed to be given by the binomial model [1] through

$$p_{partnership}=1-\left(1-p_{act}\right)^{n\tau}$$

Here $p_{act}$ is the transmission probability per coital act for the infection, $n$ is the frequency of coital acts, and $\tau$ is the smaller of the partnership duration or the infectious period of infection.

***Effective mean rate of partner change per year for sustainable transmission***

This is by definition the value of $\rho$, the effective new sexual partner acquisition rate (Protocol S1), that would render $R_{0}=1$ indicating that the infectious transmission is sustainable in the population [2].

***HIV basic reproductive number in a uniform risk behavior population***

The HIV basic reproductive number was calculated using the second generation matrix [3] to be

$$R_{0}=\frac{t_{1}\rho}{\mu+\omega_{1}}+\frac{t_{2}\rho}{\mu+\omega_{2}}\frac{\omega_{1}}{\mu+\omega_{1}}+\frac{t_{3}\rho}{\mu+\omega_{3}}\frac{\omega_{2}}{\mu+\omega_{2}}\frac{\omega_{1}}{\mu+\omega_{1}}$$

Here $t_{i}$ is the HIV transmission probability per partnership among discordant couples where the index partner is in the $i$-stage of HIV infection, $\omega_{i}$ is the progression rate from the $i$–stage to the following stage of HIV infection, and $\mu$ is the rate of removal from the sexually active population ($\mu=1/35$ corresponding to a sexually active lifespan of 35 years; the 15-49 years age group [4]).

***HSV-2 basic reproductive number in a uniform risk behavior population***

The HSV-2 basic reproductive number was calculated using the second generation matrix [3] to be

$$R_{0}=\frac{u_{1}\rho}{\mu+\pi_{1}}+\frac{u_{2}\rho\left(\mu+\pi_{3}\right)}{\mu\left(\mu+\pi_{2}+\pi_{3}\right)}\frac{\pi_{1}}{\mu+\pi_{1}}+\frac{u_{3}\rho\pi_{2}}{\mu\left(\mu+\pi_{2}+\pi_{3}\right)}\frac{\pi_{1}}{\mu+\pi_{1}}$$

Here $u_{i}$ is the HSV-2 transmission probability per partnership among discordant couples where the index partner is in the $i$-stage of HSV-2 infection and $\pi_{i}$ is the progression rate from the $i$–stage to the following stage of HSV-2 infection.

**References:**

1. Rottingen JA, Garnett GP (2002) The epidemiological and control implications of HIV transmission probabilities within partnerships. Sex Transm Dis 29: 818-827.

2. Anderson RM, May RM (1991) Infectious diseases of humans: dynamics and control. Oxford: Oxford University Press. 757 p.

3. Diekmann O, Heesterbeek JA, Metz JA (1990) On the definition and the computation of the basic reproduction ratio R0 in models for infectious diseases in heterogeneous populations. J Math Biol 28: 365-382.

4. UNAIDS/WHO *AIDS epidemic update 2007*.
