# Supplementary material for: Genital Herpes Has Played a More Important Role than Any Other Sexually Transmitted Infection in Driving HIV Prevalence in Africa
Source: PLoS One. 2008 May 21;3(5):e2230. doi: 10.1371/journal.pone.0002230 (PMC2377333; doi:10.1371/journal.pone.0002230)
Supplement: Protocol S1 — HIV and HSV-2 interaction model description. (0.06 MB PDF) [file pone.0002230.s002.pdf]

## Protocol S1

# HIV and HSV-2 interaction model description

The model consists of thirty-two coupled nonlinear ordinary differential equations that stratify the population into compartments according to HIV infection status and stage, HSV-2 infection status and stage, and the sexual risk activity class. The system of equations is an extension of conventional systems in theoretical epidemiology [1,2], and calculates the size of the epidemiologic synergy between HIV and HSV-2. The synergy is defined as the net effect of the presence of enhanced risk of HIV acquisition in HSV-2 seropositive subjects, enhanced risk of HIV transmission in dually infected persons, and enhanced HSV-2 shedding in dually infected persons.

The dynamics is described by the following system of equations:

*Fully susceptible population*

$$\frac{dS(i)}{dt} = \mu N_0(i) - \mu S(i) - \Lambda_{HIV}^{S(i)} S(i) - \Lambda_{HSV-2}^{S(i)} S(i) \tag{1}$$

*HIV infected but HSV-2 susceptible populations* $Y_\alpha(i)$

$$\begin{aligned}
\frac{dY_1(i)}{dt} &= \Lambda_{HIV}^{S(i)} S(i) - \mu Y_1(i) - \omega_{Y_1} Y_1(i) - g_{Y_1} \Lambda_{HSV-2}^{Y_1(i)} Y_1(i) \\
\frac{dY_2(i)}{dt} &= \omega_{Y_1} Y_1(i) - \mu Y_2(i) - \omega_{Y_2} Y_2(i) - g_{Y_2} \Lambda_{HSV-2}^{Y_2(i)} Y_2(i) \\
\frac{dY_3(i)}{dt} &= \omega_{Y_2} Y_2(i) - \mu Y_3(i) - \omega_{Y_3} Y_3(i) - g_{Y_3} \Lambda_{HSV-2}^{Y_3(i)} Y_3(i)
\end{aligned} \tag{2}$$

*HSV-2 seropositive but HIV susceptible populations* $I_\beta(i)$

$$\frac{dI_1(i)}{dt} = \Lambda_{HSV-2}^{S(i)} S(i) - \mu I_1(i) - \pi_{I_1} I_1(i) - h_{I_1} \Lambda_{HIV}^{I_1(i)} I_1(i)$$
$$\frac{dI_2(i)}{dt} = \pi_{I_1} I_1(i) - \mu I_2(i) - \pi_{I_2} I_2(i) - h_{I_2} \Lambda_{HIV}^{I_2(i)} I_2(i) + \pi_{I_3} I_3(i) \quad (3)$$
$$\frac{dI_3(i)}{dt} = \pi_{I_2} I_2(i) - \mu I_3(i) - \pi_{I_3} I_3(i) - h_{I_3} \Lambda_{HIV}^{I_3(i)} I_3(i)$$

*HIV and HSV-2 dually infected populations* $Z_{\alpha,\beta}(i)$

$$\frac{dZ_{1,1}(i)}{dt} = g_{Y_1} \Lambda_{HSV-2}^{Y_1(i)} Y_1(i) + h_{I_1} \Lambda_{HIV}^{I_1(i)} I_1(i) - \mu Z_{1,1}(i) - \omega_{Z_{1,1}} Z_{1,1}(i) - \pi_{Z_{1,1}} Z_{1,1}(i)$$
$$\frac{dZ_{1,2}(i)}{dt} = h_{I_2} \Lambda_{HIV}^{I_2(i)} I_2(i) + \pi_{Z_{1,1}} Z_{1,1}(i) - \mu Z_{1,2}(i) - \omega_{Z_{1,2}} Z_{1,2}(i) - \pi_{Z_{1,2}} Z_{1,2}(i) + \pi_{Z_{1,3}} Z_{1,3}(i)$$
$$\frac{dZ_{1,3}(i)}{dt} = h_{I_3} \Lambda_{HIV}^{I_3(i)} I_3(i) + \pi_{Z_{1,2}} Z_{1,2}(i) - \mu Z_{1,3}(i) - \omega_{Z_{1,3}} Z_{1,3}(i) - \pi_{Z_{1,3}} Z_{1,3}(i)$$
$$\frac{dZ_{2,1}(i)}{dt} = g_{Y_2} \Lambda_{HSV-2}^{Y_2(i)} Y_2(i) + \omega_{Z_{1,1}} Z_{1,1}(i) - \mu Z_{2,1}(i) - \omega_{Z_{2,1}} Z_{2,1}(i) - \pi_{Z_{2,1}} Z_{2,1}(i)$$
$$\frac{dZ_{2,2}(i)}{dt} = \omega_{Z_{1,2}} Z_{1,2}(i) + \pi_{Z_{2,1}} Z_{2,1}(i) - \mu Z_{2,2}(i) - \omega_{Z_{2,2}} Z_{2,2}(i) - \pi_{Z_{2,2}} Z_{2,2}(i) + \pi_{Z_{2,3}} Z_{2,3}(i)$$
$$\frac{dZ_{2,3}(i)}{dt} = \omega_{Z_{1,3}} Z_{1,3}(i) + \pi_{Z_{2,2}} Z_{2,2}(i) - \mu Z_{2,3}(i) - \omega_{Z_{2,3}} Z_{2,3}(i) - \pi_{Z_{2,3}} Z_{2,3}(i)$$
$$\frac{dZ_{3,1}(i)}{dt} = g_{Y_3} \Lambda_{HSV-2}^{Y_3(i)} Y_3(i) + \omega_{Z_{2,1}} Z_{2,1}(i) - \mu Z_{3,1}(i) - \omega_{Z_{3,1}} Z_{3,1}(i) - \pi_{Z_{3,1}} Z_{3,1}(i)$$
$$\frac{dZ_{3,2}(i)}{dt} = \omega_{Z_{2,2}} Z_{2,2}(i) + \pi_{Z_{3,1}} Z_{3,1}(i) - \mu Z_{3,2}(i) - \omega_{Z_{3,2}} Z_{3,2}(i) - \pi_{Z_{3,2}} Z_{3,2}(i) + \pi_{Z_{3,3}} Z_{3,3}(i)$$
$$\frac{dZ_{3,3}(i)}{dt} = \omega_{Z_{2,3}} Z_{2,3}(i) + \pi_{Z_{3,2}} Z_{3,2}(i) - \mu Z_{3,3}(i) - \omega_{Z_{3,3}} Z_{3,3}(i) - \pi_{Z_{3,3}} Z_{3,3}(i) \quad (4)$$

We use the notation that population variables are in capitalized Latin letters, dimensionless coefficients or factors are in small Latin letters while dimensionful quantities (such as transition rates) are in Greek symbols. The index $i$ stands for an $i$-sexual risk population where 1 represents the low-risk population while 2 represents the high risk group. The index $\alpha$ marks the stage of HIV pathogenesis; 1, 2, and 3 which stand for acute, chronic, and advanced HIV stages respectively. The index $\beta$ marks the stage of HSV-2 infection; 1, 2, and 3 which stand for

primary, latent, and activation stages respectively. Here, $N(i)$ is the population size and $N_0(i)$ is the initial population size of each $i$-risk group.

The progression of HIV is described by $\omega_{X_{\alpha=1}}$, the rate of progression from acute to chronic stage (where $X_\alpha(i)$ is any generic HIV infected population), $\omega_{X_{\alpha=2}}$, the rate from chronic to advanced stage, and $\omega_{X_{\alpha=3}}$, the rate of HIV disease mortality. The flow through HSV-2 stages is described by $\pi_{X_{\beta=1}}$, the rate of removal from primary infection to latent stage (here $X_\beta(i)$ is any generic HSV-2 infected population), $\pi_{X_{\beta=2}}$, the rate from latent stage to activation, and $\pi_{X_{\beta=3}}$, the rate from activation back to latent stage. The parameters $h_{I_\beta}$ govern the susceptibility enhancement to HIV acquisition per stage of HSV-2 infection while the parameters $g_{Y_\alpha}$ govern the susceptibility enhancement to HSV-2 acquisition per stage of HIV infection.

The rates $\Lambda_{HIV}^{Q(i)}$ and $\Lambda_{HSV-2}^{Q(i)}$ are HIV and HSV-2 forces of infection respectively as experienced by any generic susceptible population $Q(i)$. The HIV force of infection is decomposed into an HIV only infectious population and HIV-HSV-2 coinfectious population. Accordingly, the force of infection felt by the susceptible population $S(i)$ is given by $\Lambda_{HIV}^{S(i)} = \Lambda_{HIV,Y}^{S(i)} + \Lambda_{HIV,Z}^{S(i)}$ where

$$\Lambda_{HIV,Y}^{S(i)} = \rho_{S(i)} \sum_{\substack{j=1,2\\ \alpha'=1,2,3}} t_{Y_{\alpha'}(j)\to S(i)} \mathcal{G}(i,j) \frac{\rho_{Y_{\alpha'}(j)} Y_{\alpha'}(j)}{\rho_{S(j)} S(j) + \sum\limits_{\alpha''=1,2,3} \rho_{Y_{\alpha''}(j)} Y_{\alpha''}(j) + \sum\limits_{\beta''=1,2,3} \rho_{I_{\beta''}(j)} I_{\beta''}(j) + \sum\limits_{\substack{\alpha''=1,2,3\\ \beta''=1,2,3}} \rho_{Z_{\alpha'',\beta''}(j)} Z_{\alpha'',\beta''}(j)}$$

$$\Lambda_{HIV,Z}^{S(i)} = \rho_{S(i)} \sum_{\substack{j=1,2\\ \alpha'=1,2,3\\ \beta'=1,2,3}} t_{Z_{\alpha',\beta'}(j)\to S(i)} \mathcal{G}(i,j) \frac{\rho_{Z_{\alpha',\beta'}(j)} Z_{\alpha',\beta'}(j)}{\rho_{S(j)} S(j) + \sum\limits_{\alpha''=1,2,3} \rho_{Y_{\alpha''}(j)} Y_{\alpha''}(j) + \sum\limits_{\beta''=1,2,3} \rho_{I_{\beta''}(j)} I_{\beta''}(j) + \sum\limits_{\substack{\alpha''=1,2,3\\ \beta''=1,2,3}} \rho_{Z_{\alpha'',\beta''}(j)} Z_{\alpha'',\beta''}(j)}$$

(5)

And that felt by the HIV susceptible, but HSV-2 infected, population $I_\beta(i)$ is given by $\Lambda_{HIV}^{I_\beta(i)} = \Lambda_{HIV,Y}^{I_\beta(i)} + \Lambda_{HIV,Z}^{I_\beta(i)}$ where

$$\Lambda_{HIV,Y}^{I_\beta(i)} = \rho_{I_\beta(i)} \sum_{\substack{j=1,2\\ \alpha'=1,2,3}} t_{Y_{\alpha'}(j)\to I_\beta(i)} \mathscr{G}(i,j) \frac{\rho_{Y_{\alpha'}(j)} Y_{\alpha'}(j)}{\rho_{S(j)} S(j) + \sum_{\alpha''=1,2,3} \rho_{Y_{\alpha''}(j)} Y_{\alpha''}(j) + \sum_{\beta''=1,2,3} \rho_{I_{\beta''}(j)} I_{\beta''}(j) + \sum_{\substack{\alpha''=1,2,3\\ \beta''=1,2,3}} \rho_{Z_{\alpha'',\beta''}(j)} Z_{\alpha'',\beta''}(j)}$$

$$\Lambda_{HIV,Z}^{I_\beta(i)} = \rho_{I_\beta(i)} \sum_{\substack{j=1,2\\ \alpha'=1,2,3\\ \beta'=1,2,3}} t_{Z_{\alpha',\beta'}(j)\to I_\beta(i)} \mathscr{G}(i,j) \frac{\rho_{Z_{\alpha',\beta'}(j)} Z_{\alpha',\beta'}(j)}{\rho_{S(j)} S(j) + \sum_{\alpha''=1,2,3} \rho_{Y_{\alpha''}(j)} Y_{\alpha''}(j) + \sum_{\beta''=1,2,3} \rho_{I_{\beta''}(j)} I_{\beta''}(j) + \sum_{\substack{\alpha''=1,2,3\\ \beta''=1,2,3}} \rho_{Z_{\alpha'',\beta''}(j)} Z_{\alpha'',\beta''}(j)}$$

(6)

The HSV-2 force of infection is decomposed into an HSV-2 only infectious population and HIV-HSV-2 coinfectious population. Accordingly, the force of infection felt by the susceptible population $S(i)$ is given by $\Lambda_{HSV-2}^{S(i)} = \Lambda_{HSV-2,I}^{S(i)} + \Lambda_{HSV-2,Z}^{S(i)}$ where

$$\Lambda_{HSV-2,I}^{S(i)} = \rho_{S(i)} \sum_{\substack{j=1,2\\ \beta'=1,2,3}} u_{I_{\beta'}(j)\to S(i)} \mathscr{G}(i,j) \frac{\rho_{I_{\beta'}(j)} I_{\beta'}(j)}{\rho_{S(j)} S(j) + \sum_{\alpha''=1,2,3} \rho_{Y_{\alpha''}(j)} Y_{\alpha''}(j) + \sum_{\beta''=1,2,3} \rho_{I_{\beta''}(j)} I_{\beta''}(j) + \sum_{\substack{\alpha''=1,2,3\\ \beta''=1,2,3}} \rho_{Z_{\alpha'',\beta''}(j)} Z_{\alpha'',\beta''}(j)}$$

$$\Lambda_{HSV-2,Z}^{S(i)} = \rho_{S(i)} \sum_{\substack{j=1,2\\ \alpha'=1,2,3\\ \beta'=1,2,3}} u_{Z_{\alpha',\beta'}(j)\to S(i)} \mathscr{G}(i,j) \frac{\rho_{Z_{\alpha',\beta'}(j)} Z_{\alpha',\beta'}(j)}{\rho_{S(j)} S(j) + \sum_{\alpha''=1,2,3} \rho_{Y_{\alpha''}(j)} Y_{\alpha''}(j) + \sum_{\beta''=1,2,3} \rho_{I_{\beta''}(j)} I_{\beta''}(j) + \sum_{\substack{\alpha''=1,2,3\\ \beta''=1,2,3}} \rho_{Z_{\alpha'',\beta''}(j)} Z_{\alpha'',\beta''}(j)}$$

(7)

And that felt by the HSV-2 susceptible, but HIV infected, population $Y_\alpha(i)$ is given by $\Lambda_{HSV-2}^{Y_\alpha(i)} = \Lambda_{HSV-2,I}^{Y_\alpha(i)} + \Lambda_{HSV-2,Z}^{Y_\alpha(i)}$ where

$$\Lambda_{HSV-2,I}^{Y_\alpha(i)} = \rho_{Y_\alpha(i)} \sum_{\substack{j=1,2\\ \beta'=1,2,3}} u_{I_{\beta'}(j)\to Y_\alpha(i)} \mathscr{G}(i,j) \frac{\rho_{I_{\beta'}(j)} I_{\beta'}(j)}{\rho_{S(j)} S(j) + \sum_{\alpha''=1,2,3} \rho_{Y_{\alpha''}(j)} Y_{\alpha''}(j) + \sum_{\beta''=1,2,3} \rho_{I_{\beta''}(j)} I_{\beta''}(j) + \sum_{\substack{\alpha''=1,2,3\\ \beta''=1,2,3}} \rho_{Z_{\alpha'',\beta''}(j)} Z_{\alpha'',\beta''}(j)}$$

$$\Lambda_{HSV-2,Z}^{Y_\alpha(i)} = \rho_{Y_\alpha(i)} \sum_{\substack{j=1,2\\ \alpha'=1,2,3\\ \beta'=1,2,3}} u_{Z_{\alpha',\beta'}(j)\to Y_\alpha(i)} \mathscr{G}(i,j) \frac{\rho_{Z_{\alpha',\beta'}(j)} Z_{\alpha',\beta'}(j)}{\rho_{S(j)} S(j) + \sum_{\alpha''=1,2,3} \rho_{Y_{\alpha''}(j)} Y_{\alpha''}(j) + \sum_{\beta''=1,2,3} \rho_{I_{\beta''}(j)} I_{\beta''}(j) + \sum_{\substack{\alpha''=1,2,3\\ \beta''=1,2,3}} \rho_{Z_{\alpha'',\beta''}(j)} Z_{\alpha'',\beta''}(j)}$$

(8)

In these expressions, $\rho_{X(i)}$ describes the effective new sexual partner acquisition rate for each generic $X(i)$ population variable. Accordingly, sexual activity is stratified by sexual risk classes, and HIV and HSV-2 stages. Note that we use the term effective rate of partner change as opposed to rate of partner change since this parameter does not merely reflect the actual rate at which individuals change their partners, but also represents other behavioral mechanisms that effectively enhance this rate. In particular, the theoretical work on concurrency and topology of sexual networks [3,4,5,6], as well as behavioral heterogeneity [7], indicate that to a first approximation the dynamical effect of these behavioral mechanisms is to augment the rates of partner change by additional terms that effectively represent these mechanisms in the dynamics. Therefore, the partner change rates in deterministic models should not be interpreted as strictly actual partner change rates, but instead as parameters that effectively maps the hazard rate of exposure to HIV infection irrespective of the behavioral or other mechanisms that contributed to this hazard of exposure.

The mixing between the two risk groups is dictated by $\mathcal{G}(i,j)$ which is the sexual-mixing matrix that provides the probability that an individual in risk group $i$ would choose a partner in risk group $j$ [2]. It is given by the expression

$$\mathcal{G}(i,j)=e\delta_{i,j}+(1-e)\frac{\rho_{S(j)}S(j)+\sum_{\alpha'=1,2,3}\rho_{Y_{\alpha'}(j)}Y_{\alpha'}(j)+\sum_{\beta'=1,2,3}\rho_{I_{\beta'}(j)}I_{\beta'}(j)+\sum_{\substack{\alpha'=1,2,3\\ \beta'=1,2,3}}\rho_{Z_{\alpha',\beta'}(j)}Z_{\alpha',\beta'}(j)}{\sum_k\left(\rho_{S(k)}S(k)+\sum_{\alpha'=1,2,3}\rho_{Y_{\alpha'}(k)}Y_{\alpha'}(k)+\sum_{\beta'=1,2,3}\rho_{I_{\beta'}(k)}I_{\beta'}(k)+\sum_{\substack{\alpha'=1,2,3\\ \beta'=1,2,3}}\rho_{Z_{\alpha',\beta'}(k)}Z_{\alpha',\beta'}(k)\right)} \quad (9)$$

Here, $\delta_{i,j}$ is the identity matrix and the parameter $e\in[0,1]$ measures the degree of assortativeness in the mixing between the risk groups. At the extreme $e=0$, the mixing is fully proportionate (no preferential bias) while at the other extreme $e=1$, the mixing is fully assortative as individuals choose partners only from within their risk group.

The parameters $t_{R_\alpha(i)\to Q(j)}$ stand for HIV transmission probability per partnership in a partnership between any generic susceptible population $Q(j)$ and any generic HIV infected population $R_\alpha(i)$, and are expressed in terms of the HIV transmission probability per coital act in this partnership ( $p^{HIV}_{R_\alpha(i)\to Q(j)}$), the frequency of coital acts per HIV stage in this partnership ($n_{R_\alpha(i)\leftrightarrow Q(j)}$), and the duration ($\tau_{R_\alpha(i)\leftrightarrow Q(j)}$) of this partnership, using the binomial (Bernoulli) model [8]

$$t_{R_\alpha(i)\to Q(j)} = 1-\left(1-p^{HIV}_{R_\alpha(i)\to Q(j)}\right)^{n_{R_\alpha(i)\leftrightarrow Q(j)}\tau_{R_\alpha(i)\leftrightarrow Q(j)}} \tag{10}$$

The parameters $u_{R_\beta(i)\to Q(j)}$ stand for HSV-2 transmission probability per partnership in a partnership between any generic susceptible population $Q(j)$ and any generic HSV-2 infected population $R_\beta(i)$, and are expressed in terms of the HSV-2 transmission probability per coital act in this partnership ( $p^{HSV-2}_{R_\beta(i)\to Q(j)}$), the frequency of coital acts per HSV-2 stage in this partnership ($n_{R_\beta(i)\leftrightarrow Q(j)}$), and the duration ($\tau_{R_\beta(i)\leftrightarrow Q(j)}$) of this partnership, using also the binomial model

$$u_{R_\beta(i)\to Q(j)} = 1-\left(1-p^{HSV-2}_{R_\beta(i)\to Q(j)}\right)^{n_{R_\beta(i)\leftrightarrow Q(j)}\tau_{R_\beta(i)\leftrightarrow Q(j)}} \tag{11}$$

Our model formulation allows two different parameterizations of the role of enhanced HIV acquisition in HSV-2 seropositive persons. The first is by directly using the relative risk ratio ($RR$) of HIV acquisition in HSV-2 seropositive subjects to dictate the values of the $h_{I_\beta}$ parameters. Alternatively, we can derive the per-exposure cofactor ($PEC_{Acq}$) arising from the enhanced susceptibility of HSV-2 seropositive persons (Protocol S2), and use it in the expression for $t_{R_\alpha(i)\to Q(j)}$ to multiply $p^{HIV}_{R_\alpha(i)\to Q(j)}$. The availability of data determines which approach to be used. Since we were able to derive the $PEC_{Acq}$ using more than one source of data (Protocol S2), and since we cannot exclude the possibility of enhanced exposure to dually infected subjects and residual sexual-risk-behavior confounding in the available estimates for the $RR$, we have elected

here to use the per-exposure cofactor parameterization. The outcome data of the HPTN039 and Partners-in-Prevention trials [9] may allow in the future a precise estimate of the biological component in the $RR$ which would render the parameterization in terms of the $RR$ more fitting. Therefore, we have incorporated the two parameterizations in our model to allow for the potential use of each as data becomes available.

As for the demographics, we assume for simplicity stationary population size in absence of disease mortality [1]. The assumption facilitates the disentanglement of epidemiologic effects from demographic ones. Susceptibles enter the sexually active population through the rate $\mu N_0(i)$ where the natural rate of removal from the sexually active class is $\mu = 1/35$ corresponding to a sexually active lifespan of 35 years (the 15-49 years age group [10]). The value of the initial host population size depends on the community of interest. For example, in Kisumu district we have assumed a population size of 200,000 as the representative average adult population from 1980 to present in absence of HIV mortality [11,12,13].

The synergy is dictated by biological mechanisms that drives the interaction between these two pathogens (Protocol S2), and the empirical evidence suggests that these mechanisms are largely independent from sex or age (for example HSV-2 shedding frequency [14,15,16], enhanced HIV acquisition with HSV-2 seropositivity [17], and relationship between HIV viral load and HIV transmission probability [18]). Therefore, inclusion sex and age structure is unlikely to affect our estimates and conclusions for the measures of the epidemiologic synergy despite the importance of these factors in driving baseline prevalence levels (i.e. no interaction prevalences). Moreover, the best data on HIV transmission probability per coital act (the Rakai study) indicates that there are no differences between HIV transmission probability per coital from male to female and from female to male [19,20]. The sources of the disparity in HIV spread among men and women appear to be due primarily to behavioral patterns such as age cohort mixing (young women with older men) [21]. Hence to simplify the mathematical formalism we

did not include these two sources of heterogeneity and our calculations can be seen as approximate assessment of the average impact across the whole population.

It bears notice that our formalism is based on a deterministic differential equation model rather than an individual-based stochastic simulator such as in the STDSIM model [22]. Though it does not appear there are differences in the predictions due to differences in the technical methodology, our approach is amenable to an analytical examination of the dynamics that is not possible in individual-based simulation models. For example, using our approach we were able to calculate exactly and directly the $PAF$ s due to each of the mechanisms by which HSV-2 fuels HIV. Moreover, we were able to find that the major component of the dynamical role of HSV-2 in the HIV epidemic is in increasing the HIV basic reproductive number ( $R_0$ ) to be greater than 1 in part of the low-risk population thereby allowing sustainable HIV transmission in a much larger fraction of the population.

**References:**


1. Anderson RM, May RM (1991) Infectious diseases of humans: dynamics and control. Oxford: Oxford University Press. 757 p.
2. Garnett GP, Anderson RM (1993) Factors controlling the spread of HIV in heterosexual communities in developing countries: patterns of mixing between different age and sexual activity classes. Philos Trans R Soc Lond B Biol Sci 342: 137-159.
3. Kretzschmar M, Morris M (1996) Measures of concurrency in networks and the spread of infectious disease. Mathematical Biosciences 133: 165-195.
4. Morris M (1997) Sexual networks and HIV. Aids 11: S209-S216.
5. Watts CH, May RM (1992) The influence of concurrent partnerships on the dynamics of HIV/AIDS. Math Biosci 108: 89-104.
6. Morris M, Kretzschmar M (1995) Concurrent partnerships and transmission dynamics in networks. Social Networks 17: 299-318.
7. May RM, Anderson RM (1988) The Transmission Dynamics of Human Immunodeficiency Virus (Hiv). Philosophical Transactions of the Royal Society of London Series B-Biological Sciences 321: 565-607.
8. Rottingen JA, Garnett GP (2002) The epidemiological and control implications of HIV transmission probabilities within partnerships. Sex Transm Dis 29: 818-827.
9. Nagot N, Delany-Moretlwe S, Mayaud P (2007) Antiherpetic therapy for HIV infection: linking prevention and care. Future HIV Therapy 1: 131-136.
10. UNAIDS/WHO (2004) AIDS epidemic update 2004.
11. Law G (1999) Administrative Subdivisions of Countries: A Comprehensive World Reference, 1900 Through 1998 McFarland & Company 463 p.

12. Pisell TL, Hoffman IF, Jere CS, Ballard SB, Molyneux ME, et al. (2002) Immune activation and induction of HIV-1 replication within CD14 macrophages during acute Plasmodium falciparum malaria coinfection. Aids 16: 1503-1509.
13. Morison L, Weiss HA, Buve A, Carael M, Abega SC, et al. (2001) Commercial sex and the spread of HIV in four cities in sub-Saharan Africa. Aids 15 Suppl 4: S61-69.
14. Wald A, Zeh J, Selke S, Warren T, Ryncarz AJ, et al. (2000) Reactivation of genital herpes simplex virus type 2 infection in asymptomatic seropositive persons. N Engl J Med 342: 844-850.
15. Corey L, Wald A, Celum CL, Quinn TC (2004) The effects of herpes simplex virus-2 on HIV-1 acquisition and transmission: a review of two overlapping epidemics. J Acquir Immune Defic Syndr 35: 435-445.
16. Mostad SB, Kreiss JK, Ryncarz AJ, Mandaliya K, Chohan B, et al. (2000) Cervical shedding of herpes simplex virus in human immunodeficiency virus-infected women: effects of hormonal contraception, pregnancy, and vitamin A deficiency. J Infect Dis 181: 58-63.
17. Freeman EE, Weiss HA, Glynn JR, Cross PL, Whitworth JA, et al. (2006) Herpes simplex virus 2 infection increases HIV acquisition in men and women: systematic review and meta-analysis of longitudinal studies. Aids 20: 73-83.
18. Quinn TC, Wawer MJ, Sewankambo N, Serwadda D, Li CJ, et al. (2000) Viral load and heterosexual transmission of human immunodeficiency virus type 1. New England Journal of Medicine 342: 921-929.
19. Gray RH, Wawer MJ, Brookmeyer R, Sewankambo NK, Serwadda D, et al. (2001) Probability of HIV-1 transmission per coital act in monogamous, heterosexual, HIV-1-discordant couples in Rakai, Uganda. Lancet 357: 1149-1153.
20. Wawer MJ, Gray RH, Sewankambo NK, Serwadda D, Li X, et al. (2005) Rates of HIV-1 transmission per coital act, by stage of HIV-1 infection, in Rakai, Uganda. J Infect Dis 191: 1403-1409.
21. Gregson S, Nyamukapa CA, Garnett GP, Mason PR, Zhuwau T, et al. (2002) Sexual mixing patterns and sex-differentials in teenage exposure to HIV infection in rural Zimbabwe. Lancet 359: 1896-1903.
22. Freeman EE, Orroth K, White R, Glynn JR, Bakker R, et al. (2007) The proportion of new HIV infections attributable to HSV- 2 increases over time: simulations of the changing role of sexually transmitted infections in sub-Saharan African HIV epidemics. Sex Transm Infect.
