# Supplementary material for: Genital Herpes Has Played a More Important Role than Any Other Sexually Transmitted Infection in Driving HIV Prevalence in Africa
Source: PLoS One. 2008 May 21;3(5):e2230. doi: 10.1371/journal.pone.0002230 (PMC2377333; doi:10.1371/journal.pone.0002230)
Supplement: Protocol S2 — Biological and behavioral parameters. (0.11 MB PDF) [file pone.0002230.s003.pdf]

Protocol S2

Biological, behavioral and demographic parameters

HIV biological parameters

HIV transmission and progression parameters:

The HIV transmission probabilities per coital act are extracted from the measurements of Wawer *et al.* [1] by collapsing the sub-strata in their three-tier classification of incident, prevalent, and late stages into the three HIV stages: acute, chronic, and advanced, and by a reanalysis of the data using the binomial (Bernoulli) model [2] for the partnership transmission probability [3]. Hence, the partition into acute, chronic and advanced stages is adopted to represent the measurements of HIV transmission probability per stage of infection according to Wawer *et al*.

The durations of the acute and advanced stages have been chosen according to the measured probability per coital act classification in Wawer *et al.* [1], while that for the chronic stage has been determined from the measured median time from sero-conversion to death in sub-Saharan Africa [4,5] minus the time spent in the acute and advanced stages. The rates of HIV progression from one stage to the next are derived from the durations of each stage according to $\omega_i = 1/\eta_i$. Since we defined the advanced stage according to that used by Wawer *et al*. as the last two years of HIV patients before death, our definition for this stage encompasses both AIDS and over one year before the development of AIDS since the median survival from developing AIDS to death in a sub-Saharan African setting was measured to be 9.2 months [4].

The HIV-1 transmission probability per coital act varies with the HIV-1 plasma viral load according to $p_b = p_a 2.45^{\mathrm{LogIncVL}_{10}(a \to b)}$ where the $\mathrm{LogIncVL}_{10}(a \to b)$ is the logarithmic (base 10) change in the viral load from an a viral load level to a b viral load level. That is, the 2.45 factor is the rate ratio increase in transmission probability with each one-log increment in viral load [6]. We use this relation in the estimation of HIV per-exposure transmission probability from dually infected persons during HSV-2 shedding, to HIV and HSV-2 susceptible persons (biological interaction parameters section below).

HIV prevalence levels:

As for the measured HIV prevalence level in Kisumu, Kenya, there is one notable population level survey, that of the four-city study, for the duration of June 1997 to March 1998 for the 15-49 years age group [7]. There are also antenatal clinic surveillance data provided by UNAIDS for the period of 1990 to 2002 [8]. The value of these data lies in providing HIV trends since they do not necessarily reflect the HIV population prevalence level during this time period [9]. We include these data points in our calculations (Figures 1-4) and use them to fit the trend while we use the only available population survey to fit the level in the year 1997-1998 for the sexually active population [7].

HSV-2 biological parameters

HSV-2 transmission parameters:

The estimates of HSV-2 transmission probability per coital act are in the range of 0.0005 to 0.022 depending on the nature of study. Prospective partner studies predict a value of 0.0005 [10] while time to HSV-2 studies estimate it at 0.022 [11]. Considering this variation, we derived three other independent but rough estimates for this probability.

The first is calculated using the cohort data of Corey *et al*. [12] by assuming uniform and average exposure across the partnerships over the follow-up period as can be seen in Table P2 S1. The probability per coital act is calculated from the partnership probability using the binomial model ($p_{coital} = 1-(1-p_{partnership})^{1/n_{coital\ acts}}$). The number of coital acts during HSV-2 shedding is too small for a well-grounded probabilistic argument. Nevertheless, the estimates for both the placebo and treatment arms are within the confidence interval of each other. Both estimates indicate that the value of the transmission probability per coital act is at the middle of the range between prospective partner and time to HSV-2 estimates. The estimates also suggest a comparable transmission probability irrespective of the treatment status.

Table P2 S1 A rough estimate of HSV-2 transmission probability per coital act with and without valacyclovir treatment using the cohort data in Corey *et al*. [12].

	Number of people in cohort	Acquired HSV-2	Median coital acts	Partnership transmission probability during follow-up	Shedding frequency	Coital acts during HSV-2 shedding	Estimate of HSV-2 coital transmission probability
Placebo	741	27	46	27/741<br>= 0.036	10.8%	10.8%*46<br>= 4.97	0.007
Valacyclovir	742	14	49	14/743<br>= 0.019	2.9%	2.9%*49<br>= 1.42	0.013

We used the EXPLORE behavioral intervention study of men who have sex with men [13] to obtain another rough estimate also assuming uniform and average exposure across the partnerships. It was found in this study that the HSV-2 acquisition rate per 10,000 sex acts with partners of unknown HSV-2 status was 2.4 for anal intercourse with HIV seronegative partners. This estimate however includes both at-risk and not-at-risk acts, and since HSV-2 baseline prevalence among HIV seronegatives in this cohort was 20%, then only 20% of the sex acts occurred in HSV-2 discordant partnerships. Knowing that HSV-2 seropositives shed HSV-2 14% of the time [14] (see next section), then the

fraction of acts that are at risk of transmission is $20\% \times 14\% = 2.8\%$. Hence, there were 2.4 transmissions per $10,000 \times 2.8\% = 280$ at risk acts leading to a rough estimate of 0.09 for the probability of HSV-2 transmission per coital act.

A third rough estimate is derived by using our population-level model for HSV-2 spread to fit observed HSV-2 prevalence levels in populations free from a generalized HIV epidemic. By varying the HSV-2 probability per coital act and comparing the predicted prevalences to those measured, we arrive also at a coarse estimate of 0.01 for the probability per sex act.

In view of these converging estimates, it is reasonable to assume that HSV-2 transmission probability per coital act is at 0.01 with a range of 0.0005 to 0.022. The exact value used in the model is chosen by fitting the observed HSV-2 prevalence levels. Moreover, for lack of a biological mechanism, we assume that there is no HSV-2 transmission during the latent (no HSV-2 shedding) stage.

There are no measurements of the transmission probability per coital act during the primary HSV-2 infection. We assume that the transmission probability per sex act in this stage is equal to that in subsequent reactivations [15], but we assume that the primary infection lasts about twice as long as the reactivations [16]. Hence effectively the primary infection is twice as infectious as subsequent reactivations. Table P2 S3 displays our assumptions for the HSV-2 transmission probability per coital act.

HSV-2 shedding parameters:

We adopt the polymerase chain reaction (PCR) shedding data as the markers of HSV-2 infectiousness for consistency with the results that indicate ongoing transmission during periods of negative cell culture [17,18], as well as for consistency with model

predictions of HSV-2 prevalence levels. A mathematical model that constrains transmission to only the duration when there is a positive cell culture, fails to predict the observed high HSV-2 prevalence levels. Cell culture data imply much lower prevalences than are actually observed.

The rate of HSV-2 sub-clinical shedding in subjects with no reported history of genital herpes is similar to that of subjects with a known history (3.0% versus 2.7% using cell culture) [19]. Indeed, the pattern, sites, and frequency of the sub-clinical reactivation of infection are similar across persons with or with no history of symptomatic herpes infection [19]. There is also evidence that HSV-2 is often transmitted during episodes of sub-clinical shedding [20,21]. Therefore, we assume that HSV-2 seropositive persons have the same infectiousness profile regardless of the presence of clinical manifestations.

We assume an average shedding frequency of 14% of the time in HIV seronegative patients based on the state of the art measurements of HSV-2 shedding that collected anogenital swabs for HSV-2 DNA PCR at four time periods per day for 60 consecutive days [22]. The primary HSV-2 infection results in substantially longer viral shedding than subsequent reactivations. Hence, we assume that this stage lasts for 20 days; about twice the duration of that of subsequent reactivations [16,23].

HSV-2 prevalence levels:

As for the measured HSV-2 prevalence level in Kisumu, Kenya, there is one notable population level survey, that of the four-city study, for the duration of June 1997 to March 1998 for the 15-49 years age group [24]. There are no available time series for HSV-2 prevalence for the period from 1980 up to present [25,26]. However, measurements from the early nineties in three communities that are in geographic proximity to Kisumu (Rakai [27] and Masaka [28], Uganda, and Mwanza, Tanzania [29])

as well as more recent measurements in Uganda [30], indicate similar HSV-2 prevalence levels as that of Kisumu in the late nineties. Moreover, retrospective analysis of sera from Zaire suggests that HSV-2 prevalence has grown steadily since the fifties but may have saturated at its current levels by the early eighties [31]. Therefore, we assume that HSV-2 prevalence has experienced only minor variability in Kisumu since 1980. The exact level is a prediction of the model fit.

HIV and HSV-2 biological interaction parameters

Effect of dual infection on HSV-2 shedding frequency:

We assume that the HSV-2 shedding frequency in HIV infected subjects is 20% of the time for those in HIV acute and chronic stages (defined as CD4 count > 200 cells/μl) and 31% of the time in HIV advanced patients (defined as CD4 count $\leq$ 200 cells/μl). These values are derived starting from the observed shedding in HIV seronegative persons [22], and then multiplying it by the observed fractional increase in HSV-2 shedding in HIV seropositives. HIV subjects shed HSV-2 40% more while in the chronic stage, and 120% more while in the advanced stage (compare the daily HSV-2 shedding of HIV seropositive persons in [32,33] to that of HIV seronegative persons in [12]).

Though we could have used the daily HSV-2 shedding frequency in chronic and advanced HIV subjects as the average of those measured by Corey *et al*. for men (14.6% and 24.5% respectively) [33], and Mostad *et al*. for women (15.6% and 22.9% respectively) [32], these values may underestimate HSV-2 shedding since they are based on once-a-day sampling as opposed to frequent samplings per day [22]. Note that the increased shedding with HIV infection and declining CD4 cell count that we assume here is representative of the studies that measured HSV-2 shedding in HIV subjects

[32,33,34,35,36,37,38]. These estimates also indicate no substantial differences in HSV-2 shedding in dually infected persons between women and men.

Dual infection of HIV and HSV-2 impact on HIV transmissibility:

Dual infection with HIV and HSV-2 can boost HIV transmission probability per coital act through several biological mechanisms. First, there is a three to four fold (about 0.5 log base 10) increase in HIV-1 plasma viral load with dual HIV and HSV-2 infection as evidenced by several studies and proof-of-concept placebo-controlled trials that assessed the relationship between HSV-2 seropositivity and HIV viremia and measured the impact of HSV-2 suppression by acyclovir on HIV viral load [39,40,41,42,43,44,45]. This level of heightened viral load implies a 50% overall enhancement in HIV-1 transmission probability per coital act according to the functional relationship between HIV-1 plasma viral load and transmission probability per coital act [6]. Similarly, this surge in infectiousness corresponds to a factor of three increase in HIV transmission probability per coital act *during HSV-2 shedding* assuming that the surge in viremia occurs only during HSV-2 shedding.

Second, HSV family viruses has been observed to enhance HIV-1 transcription in vitro and in vivo [46,47,48,49,50], and HIV-1 RNA has been isolated with higher levels from herpetic lesions than from blood plasma [51]. Third, the clinical and sub-clinical herpetic lesions can disrupt the mucosal membranes thereby providing a portal for outgoing virons. There are however no concrete data to quantify the implications of these mechanisms on HIV-1 transmission probability per coital act. The “Partners in Prevention” study aims to shed light on this issue [52]. Earlier studies have suggested a role for dual infection in boosting HIV transmission with an overall relative risk ratio at or exceeding two for those who are dually infected with HIV and HSV-2 compared to those who are HSV-2 seronegative [53,54,55].

With these considerations in mind, we assume a three-fold increase in HIV transmission probability per sex act during HSV-2 shedding in dually infected persons based on the 0.5 log base 10 increase in HIV-1 plasma viral load with dual HIV and HSV-2 infection (that is a per-exposure cofactor effect due to enhanced transmission of $PEC_{Trans} = 3$). For the sensitivity and uncertainty analysis (Protocol S3), we vary this enhancement from 2 to 5 as a plausible range for the variation of this effect.

Susceptibility to HSV-2 infection per stage of HIV infection:

There is limited evidence that indicates changes in the susceptibility to HSV-2 infection in HIV subjects [28,56]. It has been observed that previous HIV infection is a correlate of HSV-2 sero-conversion, but it is not clear whether this observation reflects a biological increase in susceptibility versus merely residual risk-behavior confounding or an increased risk of exposure to dually infected partners who have higher HSV-2 shedding rates.

Our model results indicate that even a factor of ten enhancement has little effect on the predicted HSV-2 and HIV prevalences (<2%). The reason is that HSV-2 is much more transmissible than HIV and has a much higher prevalence. The majority of HSV-2 infections occur before acquiring HIV. Therefore, in view of the lack of sensitivity to this effect and absence of concrete evidence, we assume that there is no increased susceptibility to HSV-2 infection in HIV seropositive subjects.

Susceptibility to HIV infection per stage of HSV-2 infection:

The nature of HSV-2 infection as a leading cause of clinical and sub-clinical genital ulceration and mucosal disruption, and the presence in herpetic lesions of CD4 lymphocytes which are the HIV target cells, suggest a role for HSV-2 infection in

facilitating HIV acquisition [57,58]. This has been corroborated by numerous epidemiological studies that found a strong correlation between HSV-2 seropositivity and HIV acquisition even after controlling for sexual risk behavior. A recent meta-analysis including only longitudinal studies has determined that HSV-2 seropositive persons have an increased overall risk of HIV acquisition (RR) by a factor of 2.7 (95% confidence interval (CI),1.9-3.9) for males, and a factor of 3.1 (95% CI, 1.7-5.6) for females [59]. An earlier meta-analysis has arrived at a comparable value for the longitudinal studies of 2.1 (95% CI,1.4-3.2) for both males and females [60]. The analysis also found a risk estimate of 3.9 (95% CI, 3.1-5.1) in case-control and cross-sectional studies. In such studies, however, the temporal sequence of the two infections cannot be discerned.

The above estimates do not specify the increased risk per HSV-2 stage (primary infection and reactivation versus latent). We assume that in the latent stage there is no increase in susceptibility in view of the lack of a plausible biological mechanism. Assuming that HSV-2 seropositive persons shed the virus at a frequency of 14% of the time [22], the susceptibility enhancement *during HSV-2 shedding* has the value of 8.9 if $RR = 2.1$, and 14.6 if $RR = 2.9$ (average value for RR over males and females in [59]). Noteworthy is that the increased risk of HIV acquisition during shedding is an order of magnitude larger in value than the overall relative risk RR.

Biological per-exposure cofactor effect due to enhanced susceptibility to HIV acquisition:

We calculated the per-exposure cofactor effect (PEC) using the methodology of Hayes *et al*. [61] which links the measured overall relative risk ratio RR to the transmission probability per partnership, and subsequently to the transmission probability per coital act using the binomial (Bernoulli) model [2]. This is done by expressing the

overall relative risk of HIV acquisition in HSV-2 seropositives relative to those HSV-2 seronegative (RR) in terms of HIV transmission probability per partnership as

$$RR = \frac{z_1}{z_0} \qquad (1)$$

where z_0 is the HIV transmission probability per partnership if the HIV susceptible partner is HSV-2 seronegative

$$z_0 = 1 - (1 - p)^{n\tau_P} \qquad (2)$$

while z_1 is that if the HIV susceptible partner is HSV-2 seropositive

$$z_1 = 1 - (1 - p)^{(1-\xi_s)n\tau_P} (1 - PEC \times p)^{\xi_s n\tau_P} \qquad (3)$$

Here we assume an average HIV transmission probability per coital act of $p = 0.0015$ [1], a representative follow-up duration of HIV discordant couples of $\tau_P = 18$ months, an average coital frequency of $n = 8.9$ acts per month [54], and an HSV-2 shedding frequency in the HIV susceptible partner of $\xi_S = 14\%$ [22].

If $RR = 2.1$ [60], the above expression yields $PEC = 11.5$ indicating that the per act HIV transmission probability from an HIV seropositive person to an HSV-2 seropositive partner during the partner's HSV-2 shedding is 11.5 times larger than that of an HIV seropositive person to an HSV-2 seronegative (or latent HSV-2) partner. If we assume however an $RR = 2.9$ [59], then the $PEC = 22.2$. These estimates accord well with the early analysis of Hayes *et al.* for genital ulcers in the sense that we find the overall relative risk ratios (RR) to be much smaller than the per-exposure cofactor effect (PEC) [61]. Nevertheless, our values for the PEC , though large, are still much smaller

than what Hayes *et al.* obtained assuming only clinical ulceration and using the limited longitudinal data available at the time.

Since HSV-2 transmission probability per coital act is much larger than that of HIV, it is likely that HSV-2 is transmitted prior to HIV in any discordant partnership in both infections. It is reasonable then to assume that the RR of 2.1 [60] (or 2.9 [59]) reflect measurements in partnerships where HIV is transmitted from a dually infected person to an HSV-2 seropositive person. That is, the RR values reflect the combined effects of increased susceptibility to HIV acquisition in HSV-2 seropositive subjects as well as enhanced HIV transmissibility in dually infected patients. Therefore, we revise the above calculation by assuming that in these partnerships both partners were HSV-2 seropositive. Hence, the RR can be expressed as

$$RR = \frac{z_2}{z_0} \qquad (4)$$

where z_2 is the HIV transmission probability per partnership in a partnership of a dually infected person and an HSV-2 seropositive, but HIV seronegative, person

$$z_2 = 1 - (1-p)^{(1-\xi_s)(1-\xi_d)n\tau_P} (1 - PEC_{Acq}\, p)^{\xi_s(1-\xi_d)n\tau_P} (1 - PEC_{Trans}\, p)^{(1-\xi_s)\xi_d n\tau_P} (1 - PEC_{Acq} PEC_{Trans}\, p)^{\xi_s \xi_d n\tau_P} \qquad (5)$$

Here, PEC_{Acq} is the per-exposure cofactor arising from the enhanced susceptibility of HSV-2 seropositive persons, $PEC_{Trans} \approx 3$ is the per-exposure cofactor arising from the enhanced infectivity in dually infected persons (see subsection “Dual infection of HIV and HSV-2 impact on HIV transmissibility”), and $\xi_D = 22.2\%$ is the average HSV-2 shedding among dually infected subjects over all HIV stages (see subsection “Effect of dual infection on HSV-2 shedding frequency”). Note that $\left(1-\xi_s\right)\xi_d + \xi_s\left(1-\xi_d\right) = 30\%$ is

the fraction of the time in which there is HSV-2 shedding in one of the partners but not the other while $\xi_s \xi_d = 3\%$ is the fraction of the time in which both partners are shedding simultaneously. If $RR = 2.1$, this expression yields $PEC_{Acq} = 4.1$, while if $RR = 2.9$, the $PEC_{Acq} = 9.1$.

The seminal Rakai data provides an alternative, and independent, avenue to derive the PEC_{Acq}. It was found that the average HIV transmission probability per coital act was five fold higher with HSV-2 seropositivity (0.002 vs. 0.0004, $P = 0.01$) [33,54]. Assuming that in the partnerships where the susceptible partner was HSV-2 seropositive, the source partner was also HSV-2 seropositive, then we can derive an estimate of the PEC_{Acq} during HSV-2 shedding. The transmission probability per partnership in a partnership between a dually infected person and an HSV-2 seropositive, but HIV seronegative, person is given by

$$z_3 = 1-(1-p_0)^{(1-\xi_s)(1-\xi_d)n\tau_P}(1-PEC_{Acq}p_0)^{\xi_s(1-\xi_d)n\tau_P}(1-PEC_{Trans}p_0)^{(1-\xi_s)\xi_d n\tau_P}(1-PEC_{Acq}PEC_{Trans}p_0)^{\xi_s\xi_d n\tau_P} \quad (6)$$

Here, $p_0 = 0.0004$ is the average HIV transmission probability per coital act in absence of HSV-2 per exposure cofactor [33,54], and $\tau_P = 40$ months is the duration of follow-up in the Rakai study [1]. The z_3 probability can be also expressed as

$$z_3 = 1-(1-p_1)^{n\tau_P} \quad (7)$$

where $p_1 = 0.002$ is the average measured HIV transmission probability per coital act if the susceptible partner is HSV-2 seropositive [33,54]. Equating the expressions in Equations (6) and (7) yields a rough estimate of $PEC_{Acq} = 12.6$. This value accords well with the above estimates derived using the relative risk ratios.

Interestingly, this derived value for the PEC_{Acq} implies that the probability of HIV transmission per coital act if the susceptible partner is shedding ($PEC_{Acq}\,p_0$) is ≈ 0.005, while that when both partners are shedding ($PEC_{Acq}PEC_{Trans}\,p_0$) is ≈ 0.02. The latter value is twice as large as the measured HIV transmission probability per coital act during the HIV acute stage (defined as the first 2.5 months after HIV infection) [1]. Knowing that there is considerable evidence that indicates a large role for HIV acute stage in fueling the HIV epidemic as a consequence of the large per contact transmission probability during this stage [62,63,64,65], the strikingly large value when both partners are shedding, which happens 3% of the time, provides us with just another glimpse of how HSV-2 can easily be a culprit in fueling HIV spread.

The derivations discussed here provide merely rough estimates for the PEC_{Acq} in absence of direct measurements for this effect. Though considerably different, the PEC_{Acq} values as calculated using the RR measures found in the two meta-analyses [59,60], and the value independently calculated using the HIV transmission probabilities from the Rakai data, are broadly consistent considering the coarse-grained nature of our analyses. We adopt for our model parameters the values derived using the relative risk ratios because they reflect averages over many studies. Hence, we set $PEC_{Acq} \approx 4$ if $RR = 2.1$ [60], and $PEC_{Acq} \approx 9$ if $RR = 2.9$ [59]. Lastly, for the sensitivity and uncertainty analysis (Protocol S3) we assume a plausibility range of 3 to 10 for the PEC_{Acq} to span the plausibility range for this parameter.

Dual infection of HIV and HSV-2 impact on HSV-2 transmission probability per coital act:

Due to lack of data and absence of manifest biological mechanism, we assume that coinfection with HIV does not increase HSV-2 transmission probability per coital act.

Effect of dual infection on the natural history of HIV infection:

There is lack of evidence to show that dually infected subjects progress faster to AIDS. However, treatment with acyclovir does not seem to prolong significantly survival to AIDS among dually infected individuals [66]. Therefore, we assume for simplicity that dual infection has no effect on HIV disease progression.

Behavioral input of the model

The parameters that describe the key behavioral characteristics in Kisumu, Kenya are based on the comprehensive measurements of the four-city study [7,67,68,69,70]. A summary of these measures can be found in Table S3 in the Supporting Online Material of Abu-Raddad *et al*. [3]. We use these measures to inform the sexual behavior parameters of our model. Note that it is very complex to quantify sexual risk behavior considering the diversity of sexual behavior measures and the multitude of facets of human sexuality.

We divide the population into two sex-risk groups. The fraction of people who are in the high risk (f_{high}) versus the low-risk population is taken as the average of the following quantities: 1) proportion of men (33.5%) and of women (5.9%) who reported more than one partner (spousal or non-spousal but excluding sex workers) in the past 12 months among the sexually active population [69], 2) the proportion of men (19.5%) and of women (4.1%) with more than one non-spousal partnership (excluding contact with sex workers) in the past 12 months [68], 3) the proportion of men (3%) who had contact with female sex workers in the past 12 months [70], and 4) the number of female sex workers per man aged 15-49 years (1.95%) [68,70]. Hence we arrive at 11.3% as a representative value for the fraction of the core group in the population for both males

and females. This estimate is reasonable considering that the high risk group is a minority in the population.

We assume for simplicity that the new sexual partner acquisition rate is independent of HIV or HSV-2 infection status but depends only on the risk group status (high risk group versus the low-risk population). However, the frequency of coital acts does vary depending on HIV stage of progression as measured by Wawer *et al.* [1] and tabulated in Table P2 S4. For the new sexual partner acquisition rate among the low-risk population (ρ_{low}), we derive it based on the model fit, but using a representative value motivated by the following measures: 1) the mean number of non-spousal partners (excluding contact with sex workers) of 1.67 for men and of 1.23 for women during the last 12 months [69], 2) the average number of non-spousal partnerships (excluding contact with sex workers) for men of 701 per 1000 men-year [68], and 3) the average number of male client contacts with sex workers of 960 per 1000 men-year [68,70]. As for the new sexual partner acquisition rate among the core group (ρ_{high}), we assume also a representative value based on the model fit. The average partner change rate in the population is given by

$$\rho_{avg} = \rho_{low}\left(1 - f_{high}\right) + \rho_{high} f_{high} \qquad (8)$$

Note that the effective rate of partner change does not merely reflect the actual rate at which individuals change their partners but also represents other behavioral mechanisms that effectively enhance this rate such as concurrency and topology of sexual networks [71,72,73], and variability in risk behavior [74] (see brief discussion in Protocol S1).

There are no measurements of the assortativeness in the mixing between the risk groups in Kisumu. However, the behavior measures in [68,69,70], such as the mixing with female sex workers, suggest a limited degree of assortativeness relative to

proportionate mixing. Therefore, motivated by the model fit, we adopt a value of $e = 0.2$ for the assortativeness parameter.

The duration of sex partnerships depends on the sexual-risk group. We assume the duration of partnerships between members of the high risk group to last for 1 month. Meanwhile, partnerships in the low-risk population last for 36 months, and partnerships between a member of the high risk group and a partner in the low-risk population last for 6 months. These assumptions reflect the long duration of spousal, and to some extent non-spousal partnerships excluding contacts with sex workers (median non-spousal is 11 months in Kisumu) [68], and the variable but generally short-duration partnerships with sex workers [70].

The duration of the sexual lifespan (T) is set at 35 years to conform with the 15-49 years age groups that is typically used to define the sexually active population by the WHO as well as many HIV studies [75]. Hence, the removal rate from the sexually active population is $\mu = 1/\mathrm{T}$.

Summary of the biological, behavioral and demographic parameters

The parameters that describe HIV transmission and progression along with the per-exposure cofactors are tabulated in Table P2 S2. We assume that the per-exposure cofactors compound multiplicatively in a partnership between a dually infected index partner and an HSV-2 seropositive receiving partner. The rates of HIV progression from one stage to the next are derived from the durations of each stage according to $\omega_{Q_\alpha} = 1/\eta_\alpha$ for each population variable Q_α in HIV stage α .

Table P2 S2 Summary of the HIV transmission and progression parameters used in the model.

**Parameter**	**Value**

HIV transmission probability per coital act per stage of HIV infection:	
Acute stage ($p^{HIV}_{Y_1 \to S}$)	0.0107 [1]
Chronic stage ($p^{HIV}_{Y_2 \to S}$)	0.0008 [1]
Advanced stage ($p^{HIV}_{Y_3 \to S}$)	0.0042 [1]
Duration of each of HIV stages:	
Acute stage (η_1)	2.5 months [1]
Chronic stage (η_2)	7.59 years [4,5]
Advanced stage (η_3)	2.0 years [1]
HIV progression rates between stages:	
From acute to chronic stage ($\omega_{Y_1} = \omega_{Z_{1,\beta}}$)	4.80 per year (derived)
From chronic to advanced stage ($\omega_{Y_2} = \omega_{Z_{2,\beta}}$)	0.13 per year (derived)
From advanced stage to death ($\omega_{Y_3} = \omega_{Z_{3,\beta}}$)	0.50 per year (derived)
Rate ratio increase in HIV transmission probability per coital act per one-log (base 10) rise in viral load	2.45 [6]
Relative risk of HIV acquisition per stage of HSV-2 infection (h_{I_β})	1.0 (assumption)*
Susceptibility enhancement to HIV acquisition per-exposure cofactor during HSV-2 shedding (PEC_{Acq})	4.0 (derived based on meta-analysis in [60] for Figures 1, 3 (for the $PEC_{Acq} = 4$ calculations), 4A, 4C, and 5)

* This assumption reflects merely our choice to parameterize the effect of enhanced HIV acquisition in HSV-2 seropositive persons in terms of the acquisition per-exposure cofactor (PEC_{Acq}) rather than directly using the relative risk ratio (RR) (further discussion above on the per-exposure cofactor derivations and one comment regarding this parameterization in Protocol S1).

	9.0 (derived based on meta-analysis in [59] for Figures 2, 3 (for the $PEC_{Acq} = 9$ calculations), 4B, and 4D)
HIV infectivity enhancement per-exposure cofactor in dually infected subjects during HSV-2 shedding (PEC_{Trans})	3.0 (representative assumption based on [6,39,40,41,42,43,44])

Table P2 S3 provides a summary of our parameter choices for the HSV-2 transmission and shedding parameters. We assume that the primary HSV-2 infection lasts for 20 days irrespective of HIV status. We further assume that HSV-2 shedding occurs in the form of four reactivations per year ($\chi = 4$ per year) [16], with each cycle of HSV-2 reactivation and latency lasting for $\tau_{cycle} = \left(365/\chi\right) = 91.3$ days. Within the cycle, the duration of latency between the reactivations is given by

$$\vartheta_{I_2(Z_{\alpha,2})} = 365\left(\frac{1-\xi_{I_2(Z_{\alpha,2})}}{\chi}\right) \tag{9}$$

Meanwhile the duration of reactivation within the cycle is provided with

$$\vartheta_{I_3(Z_{\alpha,3})} = 365\left(\frac{\xi_{I_3(Z_{\alpha,3})}}{\chi}\right) \tag{10}$$

Please note how the durations of latency and reactivation depend on HIV status and stage.

Although there are substantial variations in the pattern (and frequency) of reactivations [16,22], we found in our model that the critical parameter here is the shedding frequency irrespective of whether the pattern is that of short but frequent reactivations or long but less frequent ones. This has been found by keeping the shedding

frequency fixed, but varying the pattern of shedding. The model predictions were invariable.

The rates of HSV-2 progression (π_{Q_β}) from one stage (β) to the next for any population variable Q_β are derived from the durations of each stage $\pi_{Q_\beta} = 1/\vartheta_{Q_\beta}$.

Table P2 S3 Summary of the values of HSV-2 transmission and shedding parameters in our model.

**Parameter**	**Value**
HSV-2 transmission probability per coital act per HSV-2 and HIV stage of infection:	
Primary infection $\left(p_{I_1 \to S}^{HSV-2} = p_{I_1 \to Y_{\alpha'}}^{HSV-2} = p_{Z_{\alpha,1} \to S}^{HSV-2} = p_{Z_{\alpha,1} \to Y_{\alpha'}}^{HSV-2} \right)$	0.0116 (model fit in Figures 1, 3 (for Kisumu at $PEC_{Acq} = 4$), 4A, and 4C)<br>0.0144 (model fit in Figures 2, 3 (for Kisumu at $PEC_{Acq} = 9$), 4B, and 4D)<br>0.00343 ($PEC_{Acq} = 4$) and 0.00407 ($PEC_{Acq} = 9$) (Cotonou model fit in Figure 3)<br>0.00632 ($PEC_{Acq} = 4$) and 0.00757 ($PEC_{Acq} = 9$) (Yaoundé model fit in Figure 3)<br>0.0084 ($PEC_{Acq} = 4$) and 0.01021 ($PEC_{Acq} = 9$) (Ndola model fit in Figure 3)<br>Variable transmission probability *per partnership* from 0 up to 1 (Figure 5)
Latency $\left(p_{I_2 \to S}^{HSV-2} = p_{I_2 \to Y_{\alpha'}}^{HSV-2} = p_{Z_{\alpha,2} \to S}^{HSV-2} = p_{Z_{\alpha,2} \to Y_{\alpha'}}^{HSV-2} \right)$	0 (assumption)
Reactivation $\left(p_{I_3 \to S}^{HSV-2} = p_{I_3 \to Y_{\alpha'}}^{HSV-2} = p_{Z_{\alpha,3} \to S}^{HSV-2} = p_{Z_{\alpha,3} \to Y_{\alpha'}}^{HSV-2} \right)$	0.0116 (model fit in Figures 1, 3 (for Kisumu at $PEC_{Acq} = 4$), 4A, and 4C)<br>0.0144 (model fit in Figures 2, 3 (for Kisumu at $PEC_{Acq} = 9$), 4B, and 4D)<br>0.00343 ($PEC_{Acq} = 4$) and 0.00407

	($PEC_{Acq} = 9$) (Cotonou model fit in Figure 3)<br>0.00632 ($PEC_{Acq} = 4$) and 0.00757 ($PEC_{Acq} = 9$) (Yaoundé model fit in Figure 3)<br>0.0084 ($PEC_{Acq} = 4$) and 0.01021 ($PEC_{Acq} = 9$) (Ndola model fit in Figure 3)<br>Variable transmission probability *per partnership* from 0 up to 1 (Figure 5)
HSV-2 shedding frequency among:	
HIV susceptible persons (ξ_{I_β})	14% of the time [22]
Acute HIV persons ($\xi_{Z_{1,\beta}}$)	20% of the time (derived)
Chronic HIV persons ($\xi_{Z_{2,\beta}}$)	20% of the time (derived)
Advanced HIV persons ($\xi_{Z_{3,\beta}}$)	31% of the time (derived)
Duration of the HSV-2 cycle of latency and reactivation (χ)	4 per year [16]
Duration of HSV-2 stages:	
Primary infection ($\vartheta_{I_1} = \vartheta_{Z_{\alpha,1}}$)	20.0 days (representative assumption informed by [16])
Latency between HSV-2 reactivations for HIV susceptible persons (ϑ_{I_2})	78.5 days (derived)
Latency between HSV-2 reactivations for acute HIV persons ($\vartheta_{Z_{1,2}}$)	73.0 days (derived)
Latency between HSV-2 reactivations for chronic HIV persons ($\vartheta_{Z_{3,2}}$)	73.0 days (derived)
Latency between HSV-2 reactivations for advanced HIV persons ($\vartheta_{Z_{3,2}}$)	63.0 days (derived)
Shedding during reactivation for HIV susceptible persons (ϑ_{I_3})	12.8 days (derived)

Shedding during reactivation for acute HIV persons ($\vartheta_{Z_{1,3}}$)	18.3 days (derived)
Shedding during reactivation for chronic HIV persons ($\vartheta_{Z_{3,3}}$)	18.3 days (derived)
Shedding during reactivation for advanced HIV persons ($\vartheta_{Z_{3,3}}$)	28.3 days (derived)
HSV-2 transition rates between stages:	
From primary to latent infection ($\pi_{I_1} = \pi_{Z_{\alpha,1}}$)	18.3 per year (derived)
From latent infection to reactivation among HIV susceptible persons (π_{I_2})	4.7 per year (derived)
From latent infection to reactivation among acute HIV persons ($\pi_{Z_{1,2}}$)	5.0 per year (derived)
From latent infection to reactivation among chronic HIV persons ($\pi_{Z_{2,2}}$)	5.0 per year (derived)
From latent infection to reactivation among advanced HIV persons ($\pi_{Z_{2,2}}$)	5.8 per year (derived)
From reactivation to latent infection among HIV susceptible persons (π_{I_3})	28.6 per year (derived)
From reactivation to latent infection among acute HIV persons ($\pi_{Z_{1,3}}$)	20.0 per year (derived)
From reactivation to latent infection among chronic HIV persons ($\pi_{Z_{2,3}}$)	20.0 per year (derived)
From reactivation to latent infection among advanced HIV persons ($\pi_{Z_{3,3}}$)	12.9 per year (derived)
Relative risk of HSV-2 acquisition per stage of HIV infection (g_{Y_α})	1.0 (assumption)

Table P2 S4 shows a summary of our choices for the behavioral and demographic parameters in the model. We assume an initial host population size of 200,000 in Kisumu, Kenya as the representative average adult population from 1980 to present in absence of HIV mortality [70,76,77,78].

Table P2 S4 Summary of the values of the behavioral and demographic parameters in our model.

**Parameter**	**Value**
Frequency of coital acts:	
Acute stage ($n_{Y_1(Z_{1,\beta})}$)	10.6 per month [1]
Chronic stage ($n_{Y_2(Z_{2,\beta})}$)	11.0 per month [1]
Advanced stage ($n_{Y_3(Z_{3,\beta})}$)	7.1 per month [1]
Fraction of the high risk group in the population (f_{high})	11.3% (derived based on behavioral measures in [68,69,70])
Number of people in the population at the start of the simulation	200,000 (representative assumption based on [70,76,77,78])
Number of people in the low risk group at the start of the simulation	177,400 (derived)
Number of people in the high risk group at the start of the simulation	22,600 (derived)
The new sexual partner acquisition rate:	(representative assumptions based on model fits and informed by [68,69,70])
Low-risk population (ρ_{low})	0.406 partners per year (model fit in Figures 1, 3 (at $PEC_{Acq} = 4$), 4A, and 4C)<br>0.371 partners per year (model fit in Figures 2, 3 (at $PEC_{Acq} = 9$), 4B, and 4D)<br>0.401 partners per year (model fit in Figure 5)
High risk (ρ_{high})	26.000 partners per year (model fit in Figures 1, 3 (at $PEC_{Acq} = 4$), 4A, and 4C)<br>21.000 partners per year (model fit in Figures

	2, 3 ($PEC_{Acq} = 9$), 4B, and 4D)
	30.000 partners per year (model fit in Figure 5)
Degree of assortativeness (e)	0.2 (representative assumption based on model fit and informed by [68,69,70])
Duration of sexual partnerships:	
Among the low-risk population (τ_{low})	36 months (representative assumption informed by [68,69,70])
Among the high risk population (τ_{high})	1 months (representative assumption informed by [68,69,70])
Between the low-risk and high risk populations (τ_{mixed})	6 months (representative assumption informed by [68,69,70])
Duration of the sexual lifespan (T)	35 years [75]

References:

1. Wawer MJ, Gray RH, Sewankambo NK, Serwadda D, Li X, et al. (2005) Rates of HIV-1 transmission per coital act, by stage of HIV-1 infection, in Rakai, Uganda. J Infect Dis 191: 1403-1409.
2. Rottingen JA, Garnett GP (2002) The epidemiological and control implications of HIV transmission probabilities within partnerships. Sex Transm Dis 29: 818-827.
3. Abu-Raddad LJ, Patnaik P, Kublin JG (2006) Dual infection with HIV and malaria fuels the spread of both diseases in sub-Saharan Africa. Science 314: 1603-1606.
4. Morgan D, Mahe C, Mayanja B, Okongo JM, Lubega R, et al. (2002) HIV-1 infection in rural Africa: is there a difference in median time to AIDS and survival compared with that in industrialized countries? Aids 16: 597-603.
5. Morgan D, Whitworth J (2001) The natural history of HIV-1 infection in Africa. Nat Med 7: 143-145.
6. Quinn TC, Wawer MJ, Sewankambo N, Serwadda D, Li CJ, et al. (2000) Viral load and heterosexual transmission of human immunodeficiency virus type 1. New England Journal of Medicine 342: 921-929.
7. Buve A, Carael M, Hayes RJ, Auvert B, Ferry B, et al. (2001) Multicentre study on factors determining differences in rate of spread of HIV in sub-Saharan Africa: methods and prevalence of HIV infection. Aids 15 Suppl 4: S5-14.
8. WHO/AFRO (2002) HIV/AIDS Epidemiological Surveillance Update for the WHO African Region 2002 Country Profiles.
9. UNAIDS/WHO (2003) Reconciling antenatal clinic-based surveillance and population-based survey estimates of HIV prevalence in sub-Saharan Africa.
10. Wald A, Langenberg AG, Link K, Izu AE, Ashley R, et al. (2001) Effect of condoms on reducing the transmission of herpes simplex virus type 2 from men to women. Jama 285: 3100-3106.

11. Wald A, Krantz E, Selke S, Lairson E, Morrow RA, et al. (2006) Knowledge of partners' genital herpes protects against herpes simplex virus type 2 acquisition. J Infect Dis 194: 42-52.
12. Corey L, Wald A, Patel R, Sacks SL, Tyring SK, et al. (2004) Once-daily valacyclovir to reduce the risk of transmission of genital herpes. N Engl J Med 350: 11-20.
13. Brown EL, Wald A, Hughes JP, Morrow RA, Krantz E, et al. (2006) High Risk of Human Immunodeficiency Virus in Men Who Have Sex with Men with Herpes Simplex Virus Type 2 in the EXPLORE Study. Am J Epidemiol.
14. Mark KE, Wald A, Selke S, Olin L, Huang M-L, et al. (2007) Almost Half of Genital HSV-2 Reactivations Last 6 Hours or Less. Submitted for publication.
15. Wald A (2006) Private communication.
16. Benedetti J, Corey L, Ashley R (1994) Recurrence rates in genital herpes after symptomatic first-episode infection. Ann Intern Med 121: 847-854.
17. Arvin AM, Hensleigh PA, Prober CG, Au DS, Yasukawa LL, et al. (1986) Failure of antepartum maternal cultures to predict the infant's risk of exposure to herpes simplex virus at delivery. N Engl J Med 315: 796-800.
18. Whitley R (2004) Neonatal herpes simplex virus infection. Curr Opin Infect Dis 17: 243-246.
19. Wald A, Zeh J, Selke S, Warren T, Ryncarz AJ, et al. (2000) Reactivation of genital herpes simplex virus type 2 infection in asymptomatic seropositive persons. N Engl J Med 342: 844-850.
20. Mertz GJ, Benedetti J, Ashley R, Selke SA, Corey L (1992) Risk factors for the sexual transmission of genital herpes. Ann Intern Med 116: 197-202.
21. Mertz GJ, Schmidt O, Jourden JL, Guinan ME, Remington ML, et al. (1985) Frequency of acquisition of first-episode genital infection with herpes simplex virus from symptomatic and asymptomatic source contacts. Sex Transm Dis 12: 33-39.
22. Mark KE, Wald A, Selke S, Olin L, Huang M-L, et al. (2006) Almost Half of Genital HSV-2 Reactivations Last 6 Hours or Less. Submitted for publication.
23. Ashley RL, Wald A (1999) Genital herpes: review of the epidemic and potential use of type-specific serology. Clin Microbiol Rev 12: 1-8.
24. Weiss HA, Buve A, Robinson NJ, Van Dyck E, Kahindo M, et al. (2001) The epidemiology of HSV-2 infection and its association with HIV infection in four urban African populations. Aids 15 Suppl 4: S97-108.
25. Korenromp EL, Bakker R, De Vlas SJ, Robinson NJ, Hayes R, et al. (2002) Can behavior change explain increases in the proportion of genital ulcers attributable to herpes in sub-Saharan Africa? A simulation modeling study. Sex Transm Dis 29: 228-238.
26. O'Farrell N (1999) Increasing prevalence of genital herpes in developing countries: implications for heterosexual HIV transmission and STI control programmes. Sex Transm Infect 75: 377-384.
27. Wawer MJ, Sewankambo NK, Serwadda D, Quinn TC, Paxton LA, et al. (1999) Control of sexually transmitted diseases for AIDS prevention in Uganda: a randomised community trial. Rakai Project Study Group. Lancet 353: 525-535.

28. Kamali A, Nunn AJ, Mulder DW, Van Dyck E, Dobbins JG, et al. (1999) Seroprevalence and incidence of genital ulcer infections in a rural Ugandan population. Sex Transm Infect 75: 98-102.
29. del Mar Pujades Rodriguez M, Obasi A, Mosha F, Todd J, Brown D, et al. (2002) Herpes simplex virus type 2 infection increases HIV incidence: a prospective study in rural Tanzania. Aids 16: 451-462.
30. van de Wijgert J, Morrison C, Wang J, Brown J, van der Pol B, et al. (2006) Incident HIV infections attributable to sexually transmitted and vaginal infections among women in Zimbabwe and Uganda. XVI International AIDS Conference. Toronto, Canada.
31. Nahmias AJ, Lee FK, Beckman-Nahmias S (1990) Sero-epidemiological and -sociological patterns of herpes simplex virus infection in the world. Scand J Infect Dis Suppl 69: 19-36.
32. Mostad SB, Kreiss JK, Ryncarz AJ, Mandaliya K, Chohan B, et al. (2000) Cervical shedding of herpes simplex virus in human immunodeficiency virus-infected women: effects of hormonal contraception, pregnancy, and vitamin A deficiency. J Infect Dis 181: 58-63.
33. Corey L, Wald A, Celum CL, Quinn TC (2004) The effects of herpes simplex virus-2 on HIV-1 acquisition and transmission: a review of two overlapping epidemics. J Acquir Immune Defic Syndr 35: 435-445.
34. Augenbraun M, Feldman J, Chirgwin K, Zenilman J, Clarke L, et al. (1995) Increased genital shedding of herpes simplex virus type 2 in HIV-seropositive women. Ann Intern Med 123: 845-847.
35. Hitti J, Watts DH, Burchett SK, Schacker T, Selke S, et al. (1997) Herpes simplex virus seropositivity and reactivation at delivery among pregnant women infected with human immunodeficiency virus-1. Am J Obstet Gynecol 177: 450-454.
36. Mbopi Keou FX, Gresenguet G, Mayaud P, Weiss HA, Gopal R, et al. (1999) Genital herpes simplex virus type 2 shedding is increased in HIV-infected women in Africa. Aids 13: 536-537.
37. Schacker T, Zeh J, Hu HL, Hill E, Corey L (1998) Frequency of symptomatic and asymptomatic herpes simplex virus type 2 reactivations among human immunodeficiency virus-infected men. J Infect Dis 178: 1616-1622.
38. Wald A, Corey L, Cone R, Hobson A, Davis G, et al. (1997) Frequent genital herpes simplex virus 2 shedding in immunocompetent women. Effect of acyclovir treatment. J Clin Invest 99: 1092-1097.
39. Mole L, Ripich S, Margolis D, Holodniy M (1997) The impact of active herpes simplex virus infection on human immunodeficiency virus load. J Infect Dis 176: 766-770.
40. Schacker T, Zeh J, Hu H, Shaughnessy M, Corey L (2002) Changes in plasma human immunodeficiency virus type 1 RNA associated with herpes simplex virus reactivation and suppression. J Infect Dis 186: 1718-1725.
41. Serwadda D, Gray RH, Sewankambo NK, Wabwire-Mangen F, Chen MZ, et al. (2003) Human immunodeficiency virus acquisition associated with genital ulcer disease and herpes simplex virus type 2 infection: a nested case-control study in Rakai, Uganda. J Infect Dis 188: 1492-1497.

42. Delany S, Mayaud P, Clayton T, Mlaba N, Akpomiemie G, et al. (2007) Impact of HSV-2 suppressive therapy on genital and plasma HIV-1 RNA in HIV-1 and HSV-2 seropositive women not taking anti-retroviral therapy: a randomised controlled trial in Johannesburg, South Africa, The 14th Conference on Retroviruses and Opportunistic Infections, http://www.retroconference.org/2007/.
43. Nagot N, Ouedraogo A, Foulongne V, Konate I, Weiss HA, et al. (2007) Reduction of HIV-1 RNA Levels with Therapy to Suppress Herpes Simplex Virus. N Engl J Med 356: 790-799.
44. Zuckerman RA, Lucchetti A, Whittington WLH, Sánchez J, Coombs RW, et al. (2007) HSV suppression with valacyclovir reduces rectal and blood plasma HIV-1 levels in HIV-1, HSV-2 seropositive men: A randomized, double-blind, placebo-controlled crossover trial. *under review*.
45. Baeten JM, Strick LB, Lucchetti A, Whittington WLH, J. Sanchez, et al. Herpes Simplex Virus suppressive treatment decreases plasma HIV-1 viral load in HSV-2/HIV-1 co-infected women: a randomized, placebo-controlled, cross-over trial 2007.
46. Albrecht MA, DeLuca NA, Byrn RA, Schaffer PA, Hammer SM (1989) The herpes simplex virus immediate-early protein, ICP4, is required to potentiate replication of human immunodeficiency virus in CD4+ lymphocytes. J Virol 63: 1861-1868.
47. Golden MP, Kim S, Hammer SM, Ladd EA, Schaffer PA, et al. (1992) Activation of human immunodeficiency virus by herpes simplex virus. J Infect Dis 166: 494-499.
48. Heng MC, Heng SY, Allen SG (1994) Co-infection and synergy of human immunodeficiency virus-1 and herpes simplex virus-1. Lancet 343: 255-258.
49. Mosca JD, Bednarik DP, Raj NB, Rosen CA, Sodroski JG, et al. (1987) Activation of human immunodeficiency virus by herpesvirus infection: identification of a region within the long terminal repeat that responds to a trans-acting factor encoded by herpes simplex virus 1. Proc Natl Acad Sci U S A 84: 7408-7412.
50. Mosca JD, Bednarik DP, Raj NB, Rosen CA, Sodroski JG, et al. (1987) Herpes simplex virus type-1 can reactivate transcription of latent human immunodeficiency virus. Nature 325: 67-70.
51. Schacker T, Ryncarz AJ, Goddard J, Diem K, Shaughnessy M, et al. (1998) Frequent recovery of HIV-1 from genital herpes simplex virus lesions in HIV-1-infected men. Jama 280: 61-66.
52. Nagot N, Delany-Moretlwe S, Mayaud P (2007) Antiherpetic therapy for HIV infection: linking prevention and care. Future HIV Therapy 1: 131-136.
53. Cameron DW, Simonsen JN, D'Costa LJ, Ronald AR, Maitha GM, et al. (1989) Female to male transmission of human immunodeficiency virus type 1: risk factors for seroconversion in men. Lancet 2: 403-407.
54. Gray RH, Wawer MJ, Brookmeyer R, Sewankambo NK, Serwadda D, et al. (2001) Probability of HIV-1 transmission per coital act in monogamous, heterosexual, HIV-1-discordant couples in Rakai, Uganda. Lancet 357: 1149-1153.
55. Latif AS, Katzenstein DA, Bassett MT, Houston S, Emmanuel JC, et al. (1989) Genital ulcers and transmission of HIV among couples in Zimbabwe. Aids 3: 519-523.

56. McFarland W, Gwanzura L, Bassett MT, Machekano R, Latif AS, et al. (1999) Prevalence and incidence of herpes simplex virus type 2 infection among male Zimbabwean factory workers. J Infect Dis 180: 1459-1465.
57. Cunningham AL, Turner RR, Miller AC, Para MF, Merigan TC (1985) Evolution of recurrent herpes simplex lesions. An immunohistologic study. J Clin Invest 75: 226-233.
58. Schacker T, Hu HL, Koelle DM, Zeh J, Saltzman R, et al. (1998) Famciclovir for the suppression of symptomatic and asymptomatic herpes simplex virus reactivation in HIV-infected persons. A double-blind, placebo-controlled trial. Ann Intern Med 128: 21-28.
59. Freeman EE, Weiss HA, Glynn JR, Cross PL, Whitworth JA, et al. (2006) Herpes simplex virus 2 infection increases HIV acquisition in men and women: systematic review and meta-analysis of longitudinal studies. Aids 20: 73-83.
60. Wald A, Link K (2002) Risk of human immunodeficiency virus infection in herpes simplex virus type 2-seropositive persons: a meta-analysis. J Infect Dis 185: 45-52.
61. Hayes RJ, Schulz KF, Plummer FA (1995) The cofactor effect of genital ulcers on the per-exposure risk of HIV transmission in sub-Saharan Africa. J Trop Med Hyg 98: 1-8.
62. Cohen MS, Pilcher CD (2005) Amplified HIV transmission and new approaches to HIV prevention. J Infect Dis 191: 1391-1393.
63. Jacquez JA, Koopman JS, Simon CP, Longini IM, Jr. (1994) Role of the primary infection in epidemics of HIV infection in gay cohorts. J Acquir Immune Defic Syndr 7: 1169-1184.
64. Pilcher CD, Tien HC, Eron JJ, Jr., Vernazza PL, Leu SY, et al. (2004) Brief but efficient: acute HIV infection and the sexual transmission of HIV. J Infect Dis 189: 1785-1792.
65. Abu-Raddad LJ, Longini IM, Jr. (2007) No HIV stage is dominant in driving the HIV epidemic in sub-Saharan Africa. under review.
66. Suligoi B, Dorrucci M, Volpi A, Andreoni M, Rezza G (2002) No protective effect of acyclovir on HIV disease progression in a cohort of HSV-2-HIV-infected individuals. Antivir Ther 7: 289-291.
67. Buve A, Carael M, Hayes RJ, Auvert B, Ferry B, et al. (2001) The multicentre study on factors determining the differential spread of HIV in four African cities: summary and conclusions. Aids 15 Suppl 4: S127-131.
68. Ferry B, Carael M, Buve A, Auvert B, Laourou M, et al. (2001) Comparison of key parameters of sexual behaviour in four African urban populations with different levels of HIV infection. Aids 15 Suppl 4: S41-50.
69. Lagarde E, Auvert B, Carael M, Laourou M, Ferry B, et al. (2001) Concurrent sexual partnerships and HIV prevalence in five urban communities of sub-Saharan Africa. Aids 15: 877-884.
70. Morison L, Weiss HA, Buve A, Carael M, Abega SC, et al. (2001) Commercial sex and the spread of HIV in four cities in sub-Saharan Africa. Aids 15 Suppl 4: S61-69.
71. Kretzschmar M, Morris M (1996) Measures of concurrency in networks and the spread of infectious disease. Mathematical Biosciences 133: 165-195.

72. Morris M (1997) Sexual networks and HIV. Aids 11: S209-S216.
73. Watts CH, May RM (1992) The influence of concurrent partnerships on the dynamics of HIV/AIDS. Math Biosci 108: 89-104.
74. May RM, Anderson RM (1988) The Transmission Dynamics of Human Immunodeficiency Virus (Hiv). Philosophical Transactions of the Royal Society of London Series B-Biological Sciences 321: 565-607.
75. UNAIDS/WHO *AIDS epidemic update 2005* (available at http://www.unaids.org/epi/2005/doc/report_pdf.asp, accessed 11 April 2006).
76. Law G (1999) Administrative Subdivisions of Countries: A Comprehensive World Reference, 1900 Through 1998 McFarland & Company 463 p.
77. Law G (2005) Provinces of Kenya, Kenya Central Bureau of Statistics, Administrative Divisions of Countries ("Statoids"), http://www.statoids.com/uke.html.
78. Law G (2005) Districts of Kenya, Kenya Central Bureau of Statistics, Administrative Divisions of Countries ("Statoids"), http://www.statoids.com/yke.html.
