# Supplementary material for: Genital Herpes Has Played a More Important Role than Any Other Sexually Transmitted Infection in Driving HIV Prevalence in Africa
Source: PLoS One. 2008 May 21;3(5):e2230. doi: 10.1371/journal.pone.0002230 (PMC2377333; doi:10.1371/journal.pone.0002230)
Supplement: Protocol S3 — Uncertainty and sensitivity analyses of the key parameters in the model. (0.57 MB DOC) [file pone.0002230.s004.doc]

**Protocol S3**

**Uncertainty and sensitivity analyses of the key parameters and basic assumptions in the model**

**Uncertainty and sensitivity analyses of the key parameters in the model**

We examined the sensitivity of our predictions for the five measures of the epidemiologic synergy discussed in this article to various parameters in the model. We did so by conducting sensitivity and uncertainty analyses through Monte Carlo sampling from specified ranges of uncertainty for the key parameters. We sampled from the specified ranges using the uniform distribution for 1000 runs of the model for each parameter. The parameters and ranges of variation are 3 to 10 for the $PEC_{Acq}$ (per-exposure cofactor effect due to enhanced susceptibility of HSV-2 seropositive persons), 2 to 5 for the $PEC_{Trans}$ (per-exposure cofactor effect due to enhanced infectivity of dually infected persons), $\pm$15 for the HSV-2 shedding frequency, $\pm$15 for the HSV-2 transmission probability per coital act, $\pm$15 for the new sexual partner acquisition rate, and $\pm$15 for the fraction of the population in the high risk group. These choices represent the most influential parameters in our formalism as seen in multiple analyses of the model and there are not precise estimates for them. The ranges of variation represent plausible values for these parameters given the empirical evidence discussed in Protocol S2.

The analyses for the HIV and HSV-2 excess prevalences were calculated by subtracting the mean, mean plus standard deviation, and mean minus standard deviation in 1000 runs of the model assuming a scenario where there is no interaction between the two pathogens from the corresponding quantities assuming a scenario where there is an interaction between the two diseases. Note that the purpose of these sensitivity analyses is not strictly to establish the sensitivity of different variables, such as baseline prevalences (prevalences in absence of interaction), to certain parameters in the model, but to examine whether the uncertainty in our knowledge of some of the parameters affect our predictions specifically for the size of the epidemiological synergy as expressed in the five measures of $PAF_{Sus}$, $PAF_{Inf}$, $PAF_{Sus+Inf}$, and HIV and HSV-2 excess prevalences.

Figure P3S1 shows the results of the sensitivity and uncertainty analyses for $PAF_{Sus}$, $PAF_{Inf}$, $PAF_{Sus+Inf}$, and HIV and HSV-2 excess prevalences as calculated in the year of 1997 (when the four-city study was conducted [1]) for the model fit of Figure 1 of the main text. The size of the synergy is primarily sensitive to the values of the $PEC_{Acq}$, $PEC_{Trans}$, and HSV-2 shedding frequency (when the per-exposure cofactors are acting), but not to the HSV-2 transmission probability per coital act, new sexual partner acquisition rate, and fraction of the population in the high risk group. Note that our derivation of the HIV acquisition per-exposure cofactor $PEC_{Acq}$ (Protocol S2) also depends on the value of the HSV-2 shedding frequency.

These results indicate how the magnitude of the epidemiologic synergy between HIV and HSV-2 is primarily and directly sensitive to the interaction parameters that dictate the strength of the interaction between the two diseases. The $PEC_{Acq}$ and $PEC_{Trans}$ play the leading role in determining the magnitude of the epidemiological synergy. A precise assessment of these parameters is a necessity before any conclusive and quantitatively robust assessment of HSV-2 role in fueling the HIV epidemic can be discerned. However other parameters, such as the behavioral parameters, though strongly influence the baseline prevalence levels of the two diseases, they do not directly affect the synergy. Baseline prevalence levels can be used as proxies of the indirect impact of HIV and HSV-2 baseline (no interaction) infectious spread parameters. A proper quantitative characterization of the two diseases interaction parameters, and the two diseases prevalences and their overlap, is the key determinant in predicting the ramifications of the interaction.


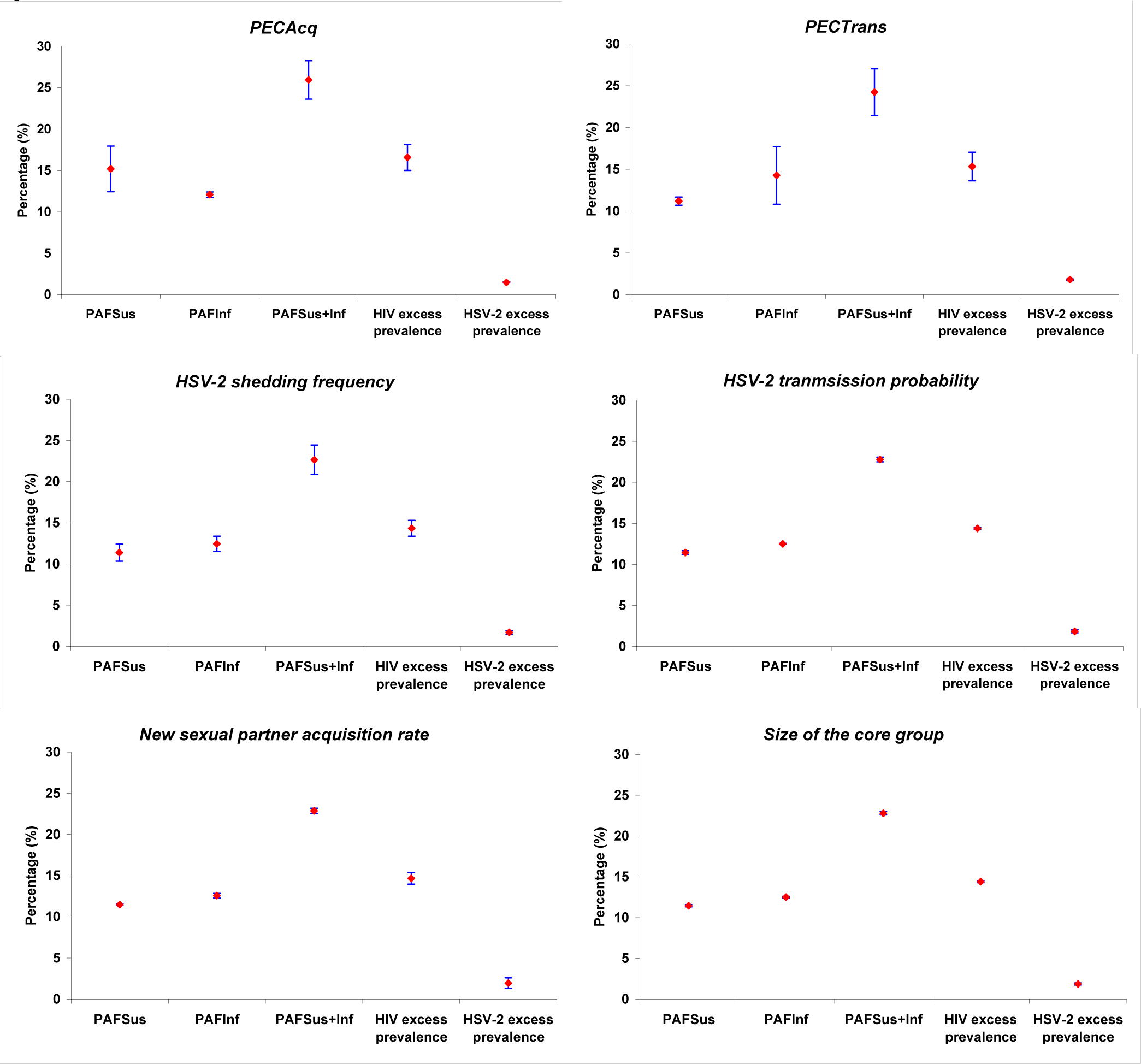


Figure P3 S1 Results of the sensitivity and uncertainty analyses. The sensitivity of the epidemiologic synergy measures of $PAF_{Sus}$, $PAF_{Inf}$, $PAF_{Sus+Inf}$, and HIV and HSV-2 excess prevalences as calculated in the year of 1997 for the calculation of Figure 1 of the main text. The sensitivity is calculated with respect to the parameters of $PEC_{Acq}$ (per-exposure cofactor effect due to enhanced susceptibility of HSV-2 seropositive persons), $PEC_{Trans}$ (per-exposure cofactor effect due to enhanced infectivity of dually infected persons), HSV-2 shedding frequency, HSV-2 transmission probability per coital act, new sexual partner acquisition rate, and the fraction of the population in the high risk group. The calculations were done by Monte Carlo sampling from specified ranges of uncertainty for the parameters using the uniform distribution for 1000 runs of the model for each parameter.

**Epidemiologic synergy as a function of susceptibility and infectivity enhancements**

We discus here how the epidemiologic synergy depends functionally on $PEC_{Acq}$ and $PEC_{Trans}$. Figure P4 S2 shows the epidemiologic synergy measures as functions of the $PEC_{Acq}$ while Figure P4 S3 displays the same measures as functions of the $PEC_{Trans}$. The calculations were done at the endemic equilibrium to disentangle the interaction-strength effects from the temporal effects. The parameters of the model fits are the same as those of Figure 5 of the main text, but with $p^{HSV-2}=0.0172$.

Although both the $PEC_{Acq}$ and $PEC_{Trans}$ strongly drive the epidemiologic synergy, the value of the $PEC_{Trans}$ is more impactful than an equal value for the$PEC_{Acq}$. This is a consequence of the fact that the $PEC_{Trans}$ effect occurs in dually infected subjects who shed HSV-2 substantially more frequently than HSV-2 only infected persons where the $PEC_{Acq}$ effect is acting.


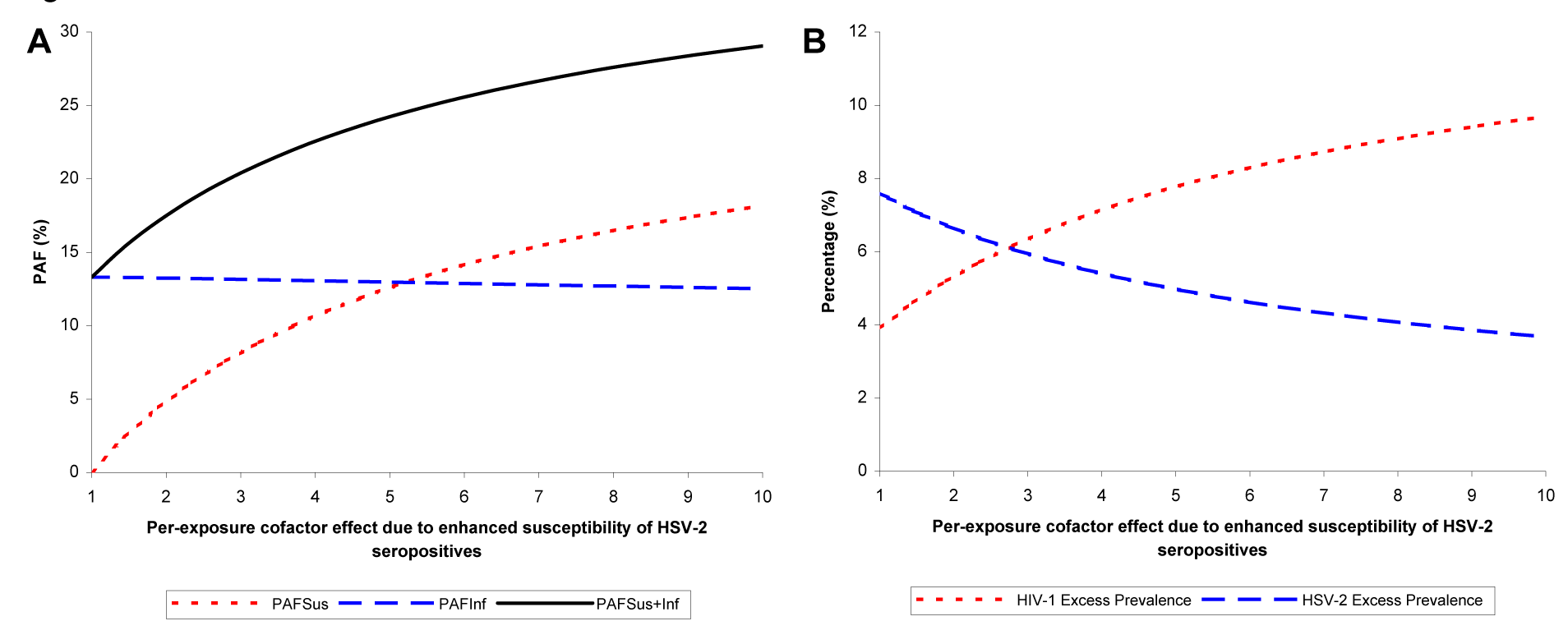


Figure P3 S2 Impact of enhanced risk of HIV acquisition in HSV-2 seropositive persons ($PEC_{Acq}$) on the epidemiologic synergy. The left panel (A) shows $PAF_{Sus}$, $PAF_{Inf}$, and $PAF_{Sus+Inf}$. The right panel (B) displays HIV and HSV-2 excess prevalences. All variables are shown as functions of the $PEC_{Acq}$. The HIV and HSV-2 prevalences in absence of interaction are at 20% and 40% respectively. The $PEC_{Trans}=3$.


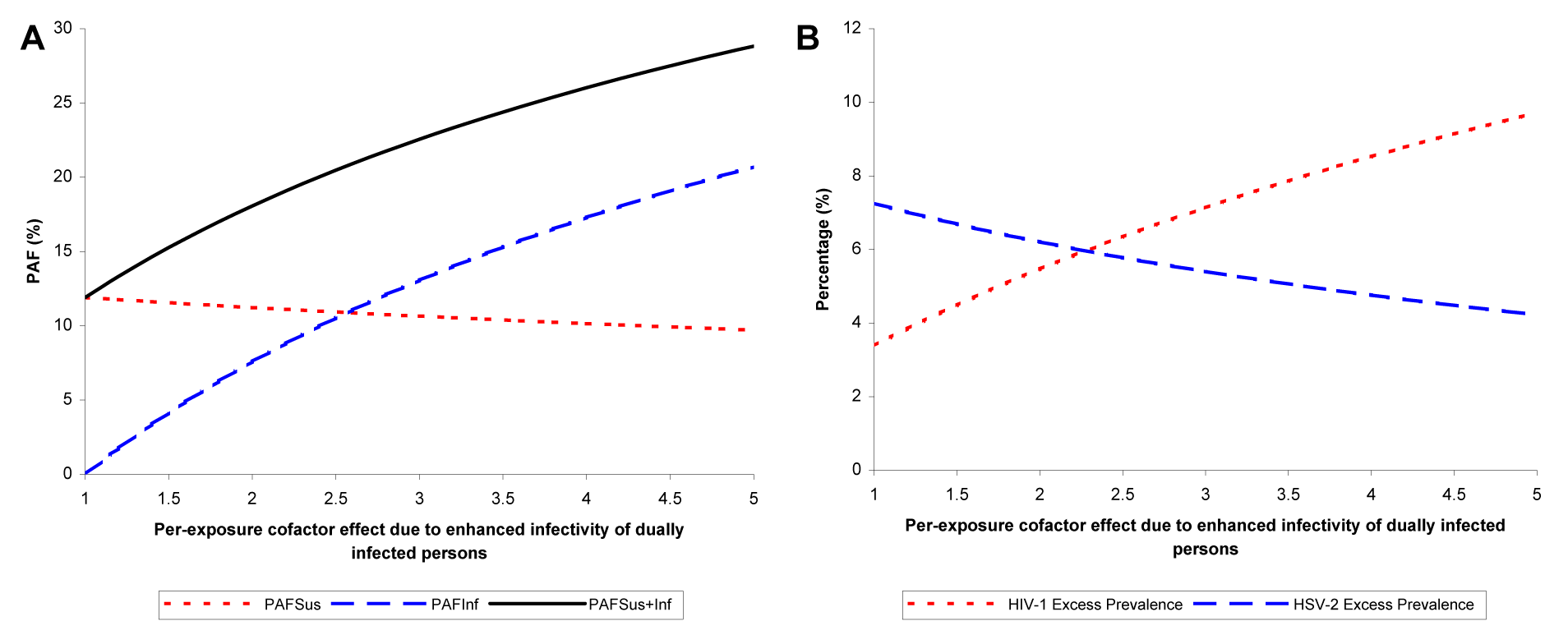


Figure P3 S3 Impact of enhanced HIV infectivity in dually infected persons ($PEC_{Trans}$) on the epidemiologic synergy. The left panel (A) shows $PAF_{Sus}$, $PAF_{Inf}$, and $PAF_{Sus+Inf}$. The right panel (B) displays HIV and HSV-2 excess prevalences. All variables are shown as functions of the$PEC_{Trans}$. The HIV and HSV-2 prevalences in absence of interaction are at 20% and 40% respectively. The $PEC_{Acq}=4$.

**Competing risks and the role of other sexually transmitted infections**

There are multiple risks for HIV acquisition and transmission and different biological cofactors can impact the HIV dynamics. In this work we focused on the role of one biological cofactor, namely genital herpes, and there is a question as to whether the presence of other sexually transmitted infections (STIs), such as ulcerative bacterial STIs, can influence our assessment of the $PAF$s due to HSV-2 through the dynamics of competing risks. To address this question, we conducted a calculation using a simplified version of our model that includes a bacterial ulcerative STI cofactor as well as an HSV-2 cofactor to examine whether the lack of inclusion of such STIs can make a substantial difference.

The model consists of 12 differential equations that stratify the population into compartments according to HIV infection status and stage, HSV-2 cofactor status, ulcerative bacterial STI cofactor status, and the sexual risk activity class:

$$\begin{matrix} \frac{dS(i)}{dt}=\left(1-f_{HSV-2}-f_{BSTI}\right)\mu N_{0}(i)-\mu S(i)-\Lambda_{HIV}^{S(i)}S(i) \\ \frac{dH(i)}{dt}=f_{HSV-2}\mu N_{0}(i)-\mu H(i)-RR(i)_{HSV-2}\Lambda_{HIV}^{S(i)}H(i) \\ \frac{dB(i)}{dt}=f_{BSTI}\mu N_{0}(i)-\mu B(i)-RR(i)_{BSTI}\Lambda_{HIV}^{S(i)}B(i) \\ \frac{dY_{1}(i)}{dt}=\Lambda_{HIV}^{S(i)}S(i)+RR(i)_{HSV-2}\Lambda_{HIV}^{S(i)}H(i)+RR(i)_{BSTI}\Lambda_{HIV}^{S(i)}B(i)-\mu Y_{1}(i)-\omega_{Y_{1}}Y_{1}(i) \\ \frac{dY_{2}(i)}{dt}=\omega_{Y_{1}}Y_{1}(i)-\mu Y_{2}(i)-\omega_{Y_{2}}Y_{2}(i) \\ \frac{dY_{3}(i)}{dt}=\omega_{Y_{2}}Y_{2}(i)-\mu Y_{3}(i)-\omega_{Y_{3}}Y_{3}(i) \end{matrix}$$

Here, $S(i)$, $H(i)$, and $B(i)$ are the susceptible, HSV-2 seropositive but HIV susceptible, and ulcerative bacterial STI infected but HIV susceptible populations respectively. The $Y_{\alpha}(i)$ are the HIV infected populations in stage $\alpha$. The $RR(i)_{HSV-2}$ and $RR(i)_{BSTI}$ are the parameters that dictate the enhanced risk to HIV acquisition due to the presence of the biological cofactors of HSV-2 seropositivity or ulcerative bacterial STI infection respectively, and $f_{HSV-2}$ and $f_{BSTI}$ are the fractions of the population that have these risk factors. The index $i$ marks the sexual risk activity class. The rest of the parameters are similar to those defined in the HIV and HSV-2 model in Protocol S1.

Figure P3S4 shows the result of the calculation under the assumption that the high risk group has a total bacterial ulcerative STI prevalence of 20% and the low risk group has a prevalence of 2%. HSV-2 prevalence is assumed 50% in the whole population. We further assume that the relative risk of HIV acquisition due to the presence of the bacterial ulcerative STI cofactor is equal to that of HSV-2 at $RR(i)_{BSTI}=RR(i)_{HSV-2}=2$. Note that this simulation assumes even a substantially higher bacterial ulcerative STI prevalence than is actually measured [2] just to highlight our point. Indeed, the prevalence of bacterial STIs is very limited in the low risk population and is generally in sharp decline in sub-Saharan Africa [3,4,5,6,7]. The results show the predictions if we include both bacterial and HSV-2 cofactors as compared to the analysis with only the HSV-2 cofactor. The presence of the bacterial STIs reduced the $PAF$ due to HSV-2 in the high risk group from 33% to 29%, and has virtually no impact on the $PAF$ in the low risk group. Even though bacterial STIs contributed in this risk group substantially to the HIV epidemic ($PAF$ of HIV due to bacterial ulcerative STI is 12%), the dynamics of competing risks reduced the $PAF$ due to HSV-2 by only 4%. The only assumptions by which bacterial STIs could affect our calculations, and only in the high risk group, is with an excessively very high per-exposure cofactor and high prevalence for bacterial ulcerative STIs, which is not supported by empirical data [8].


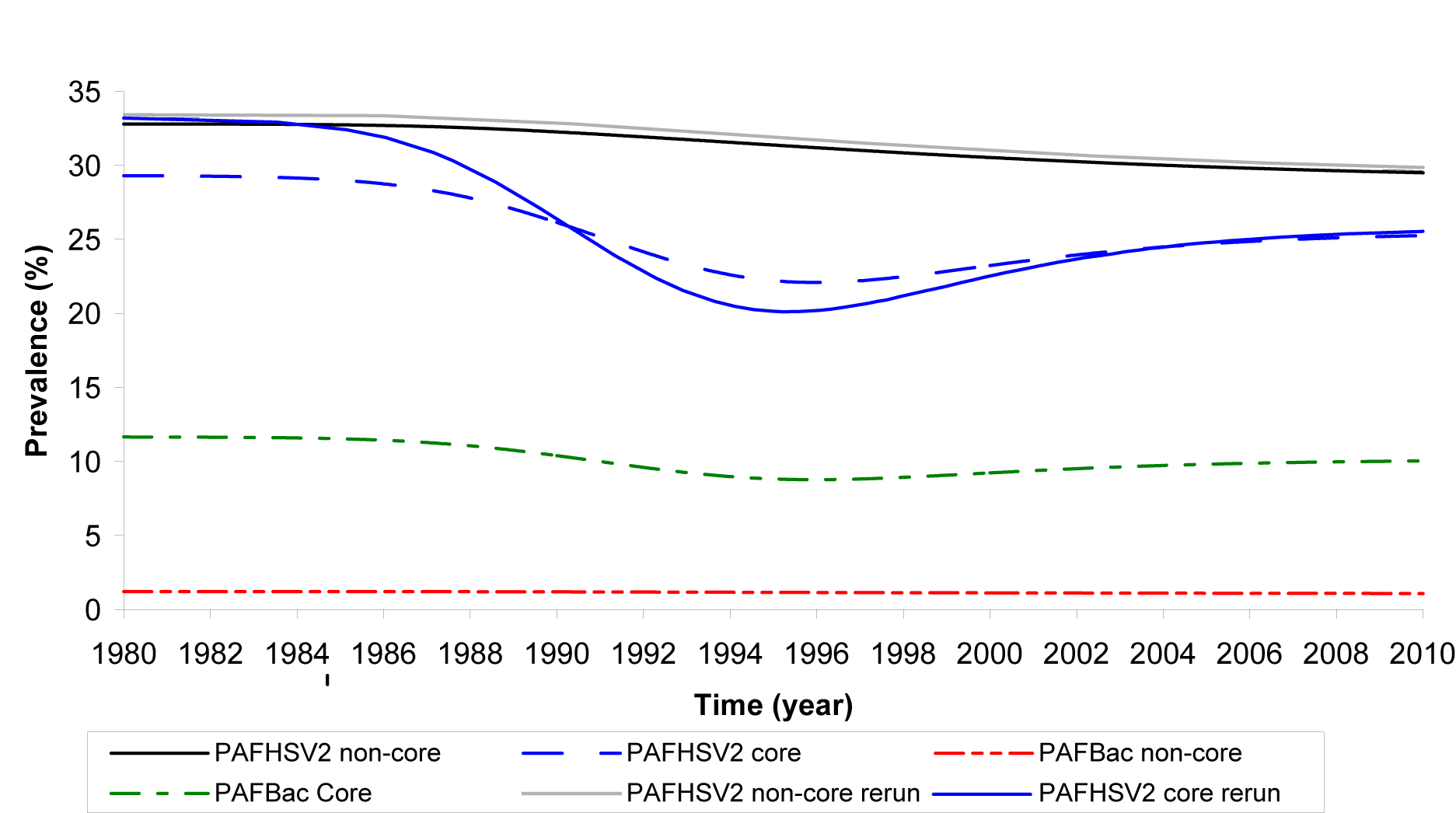


Figure P4 S4 Impact of competing risks between HSV-2 role and ulcerative bacterial STIs role in HIV dynamics. The $PAF$ due to HSV-2 per risk group as calculated in a model that includes ulcerative bacterial STIs cofactor compared to the $PAF$ due to HSV-2 per risk group in a model that does not include this cofactor. The figure also shows the $PAF$ due to ulcerative bacterial STIs per risk group.

**References**

1. Buve A, Carael M, Hayes RJ, Auvert B, Ferry B, et al. (2001) Multicentre study on factors determining differences in rate of spread of HIV in sub-Saharan Africa: methods and prevalence of HIV infection. Aids 15 Suppl 4: S5-14.

2. Buve A, Carael M, Hayes RJ, Auvert B, Ferry B, et al. (2001) The multicentre study on factors determining the differential spread of HIV in four African cities: summary and conclusions. Aids 15 Suppl 4: S127-131.

3. Korenromp EL, Bakker R, De Vlas SJ, Robinson NJ, Hayes R, et al. (2002) Can behavior change explain increases in the proportion of genital ulcers attributable to herpes in sub-Saharan Africa? A simulation modeling study. Sex Transm Dis 29: 228-238.

4. Korenromp EL, Bakker R, de Vlas SJ, Gray RH, Wawer MJ, et al. (2002) HIV dynamics and behaviour change as determinants of the impact of sexually transmitted disease treatment on HIV transmission in the context of the Rakai trial. Aids 16: 2209-2218.

5. Robinson NJ, Mulder DW, Auvert B, Hayes RJ (1997) Proportion of HIV infections attributable to other sexually transmitted diseases in a rural Ugandan population: simulation model estimates. Int J Epidemiol 26: 180-189.

6. Kaul R, Kimani J, Nagelkerke NJ, Fonck K, Ngugi EN, et al. (2004) Monthly antibiotic chemoprophylaxis and incidence of sexually transmitted infections and HIV-1 infection in Kenyan sex workers: a randomized controlled trial. Jama 291: 2555-2562.

7. Korenromp EL, Van Vliet C, Grosskurth H, Gavyole A, Van der Ploeg CP, et al. (2000) Model-based evaluation of single-round mass treatment of sexually transmitted diseases for HIV control in a rural African population. Aids 14: 573-593.

8. Gray RH, Serwadda D, Wawer MJ (2007) Empirical observations underestimate the proportion of HIV infections attributable to sexually transmitted diseases in Mwanza and Rakai STD treatment trials: simulation results. Sex Transm Dis 34: 61; author reply 62.
